# Supplementary material for: Identification of biomarkers for glycaemic deterioration in type 2 diabetes
Source: Nat Commun. 2023 May 3;14:2533. doi: 10.1038/s41467-023-38148-7 (PMC10156700; doi:10.1038/s41467-023-38148-7)
Supplement: Supplementary file 1 — Supplementary Information [file 41467_2023_38148_MOESM1_ESM.pdf]

SUPPLEMENTARY FIGURES AND TABLES

Belonging to Slieker, Donnelly and Akalestou *et al.* **Identification of biomarkers for glycaemic deterioration in type 2 diabetes**

SUPPLEMENTAL FIGURE 1. FLOWCHART OF THE CURRENT STUDY. 2

SUPPLEMENTAL FIGURE 2. MEDIAN SCALED LEVELS OF THE TOP METABOLITES ACROSS THE THREE DISCOVERY COHORTS. 3

SUPPLEMENTAL FIGURE 3. MEDIAN SCALED LEVELS OF THE TOP LIPIDS ACROSS THE THREE DISCOVERY COHORTS. 4

SUPPLEMENTAL FIGURE 4. RELATION BETWEEN THE HAZARD RATIO OF TIME TO INSULIN INITIATION VERSUS THE ACYL CHAIN LENGTH 5

SUPPLEMENTAL FIGURE 5. CORRELATION BETWEEN THE TOP IDENTIFIED BIOMARKERS IN ACROSS THE THREE COHORTS 6

SUPPLEMENTAL FIGURE 6. MEDIAN SCALED LEVELS OF THE TOP PROTEINS ACROSS THE TWO DISCOVERY COHORTS (DCS, GODARTS). 7

SUPPLEMENTAL FIGURE 7. THE TUNEL STAINING OF HUMAN PANCREATIC ISLETS TREATED WITH CYTOKINES AND RECOMBINANT HUMAN NOGOR PROTEIN. 8

SUPPLEMENTARY FIGURE. 8. EFFECTS OF NOGOR ON INSULIN-STIMULATED PHOSPHORYLATION OF INSULIN RECEPTOR AND AKT IN PRIMARY MOUSE HEPATOCYTES. 10

SUPPLEMENTARY FIGURE 9. EFFECTS OF NOGOR ON INSULIN-STIMULATED PHOSPHORYLATION OF AKT IN ADIPOCYTES DERIVED FROM C3H10T1/2 CELLS. 12

SUPPLEMENTARY FIGURE 10. EFFECTS OF NOGOR AND CRELD1 ON INSULIN-STIMULATED PHOSPHORYLATION OF AKT IN HEPG2 CELLS. 14

TABLE S1 CHARACTERISTICS OF THE INCLUDED DISCOVERY AND VALIDATION COHORTS 15

TABLE S2 COX PROPORTIONAL HAZARD RATIO OF BASE MODELS WITHOUT BIOMARKERS. 16

TABLE S3 COX PROPORTIONAL HAZARD MODELS FOR THE METABOLITES 17

TABLE S4. PQTLS ASSOCIATED WITH TOP APTAMERS. 18

LIPIDOMICS MINIMAL REPORTING CHECKLIST 19

SOURCE DATA (UNCROPPED GELS) 20

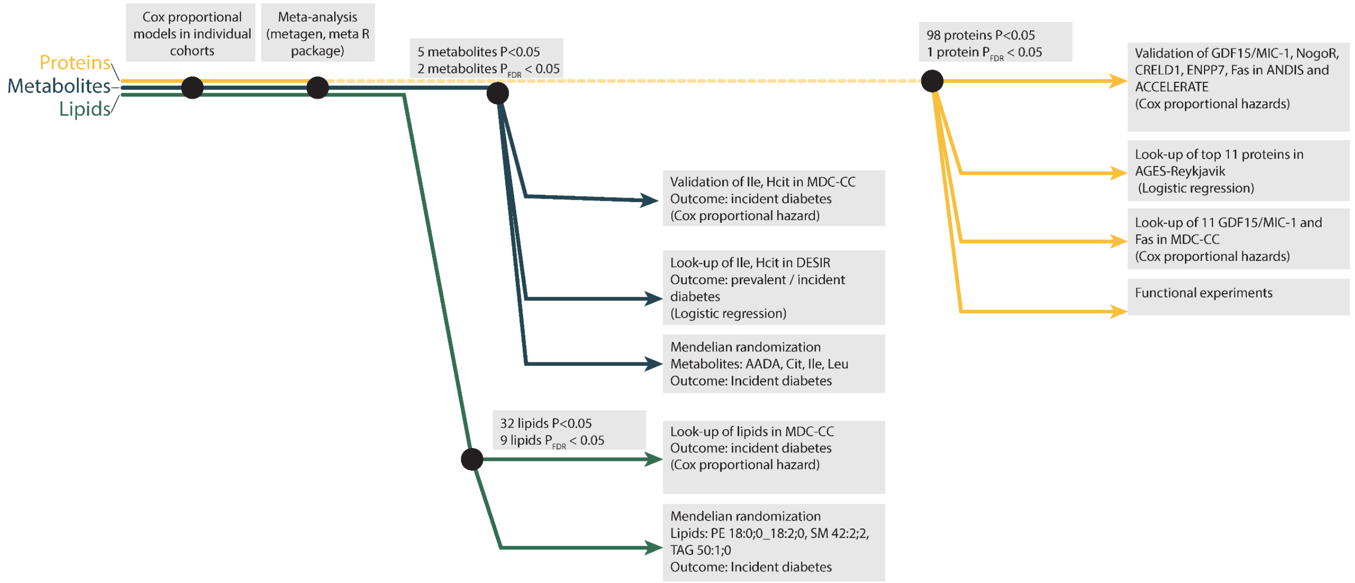

Supplemental Figure 1. Flowchart of the current study. Statistical test for discovery: Cox proportional hazard model.

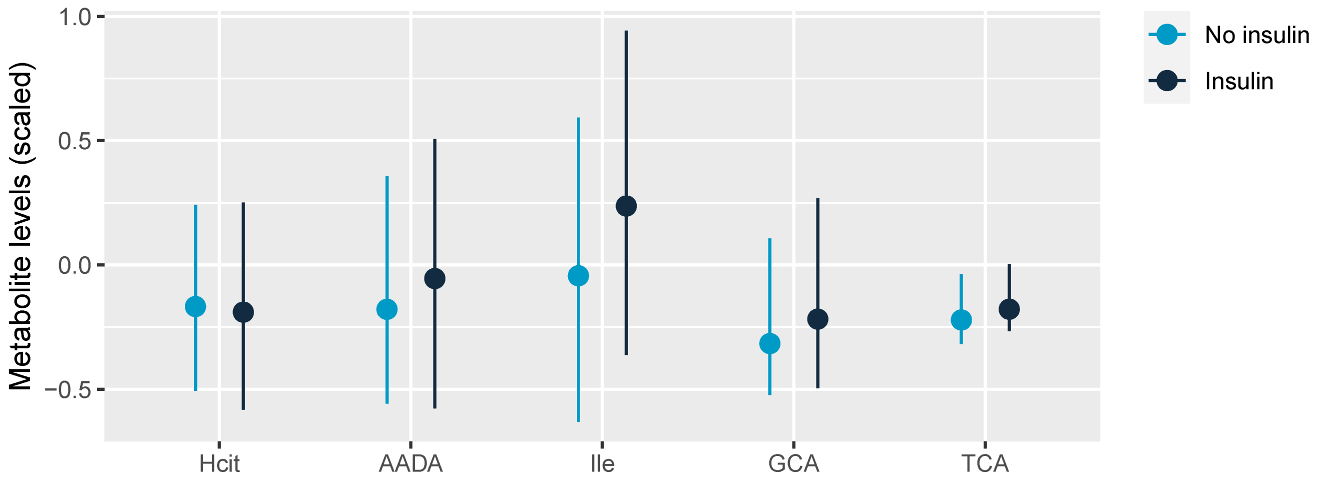

Supplemental Figure 2. Median scaled levels of the top metabolites across the three discovery cohorts. Light blue no incident insulin use, dark blue, incident insulin use. Data are presented as median with 25% and 75% quantile across the three cohorts.

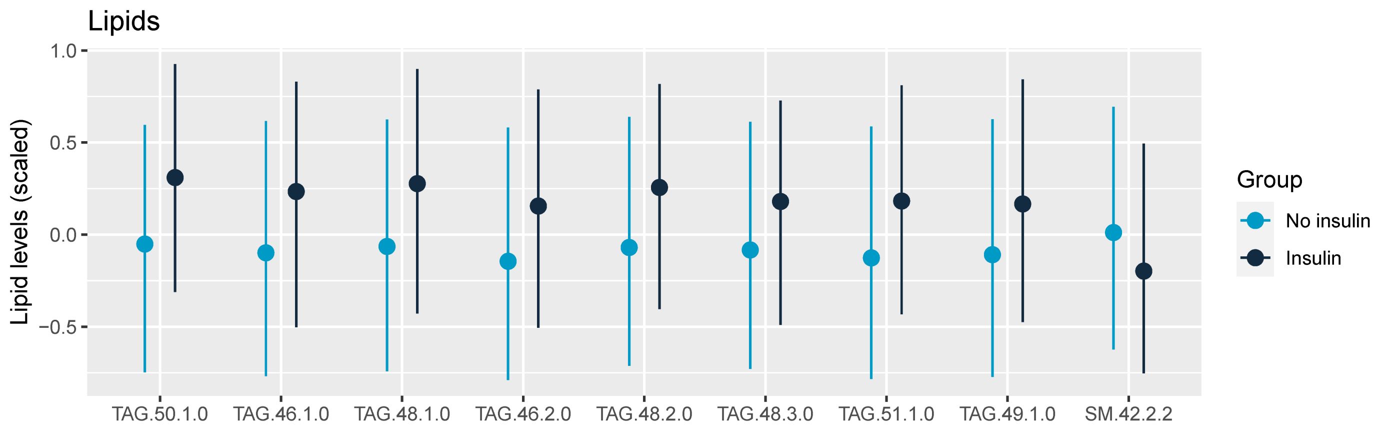

Supplemental Figure 3. Median scaled levels of the top lipids across the three discovery cohorts. Light blue no incident insulin use, dark blue, incident insulin use. Data are presented as median with 25% and 75% quantile across the three cohorts.

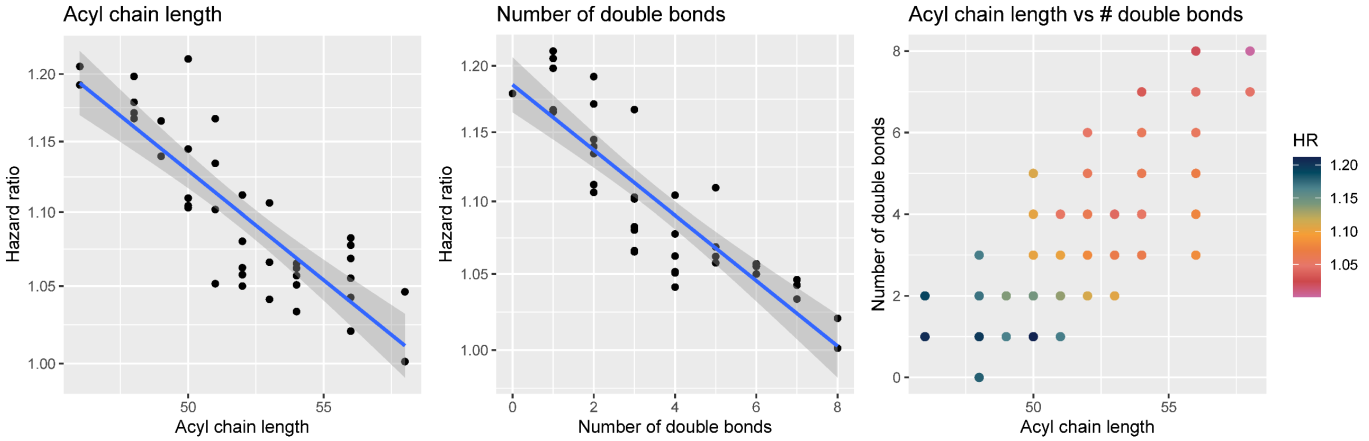

Supplemental Figure 4. Relation between the hazard ratio of time to insulin initiation versus the acyl chain length (a) and number of double bonds (b) and their relation (c). Blue line represents a linear regression line with 95% confidence interval.

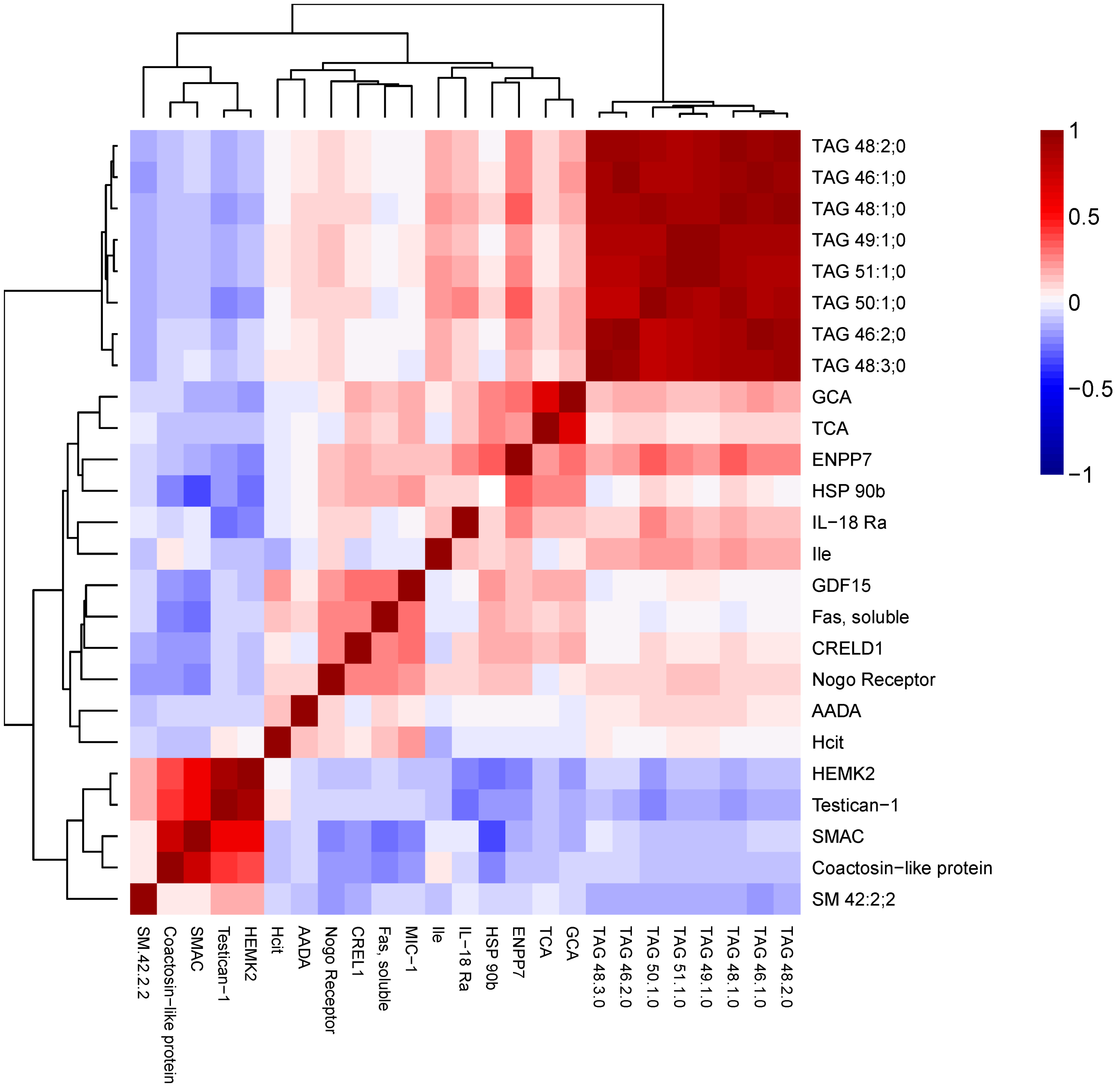

Supplemental Figure 5. Correlation between the top identified biomarkers in across the three cohorts (two cohorts in case of the proteins).

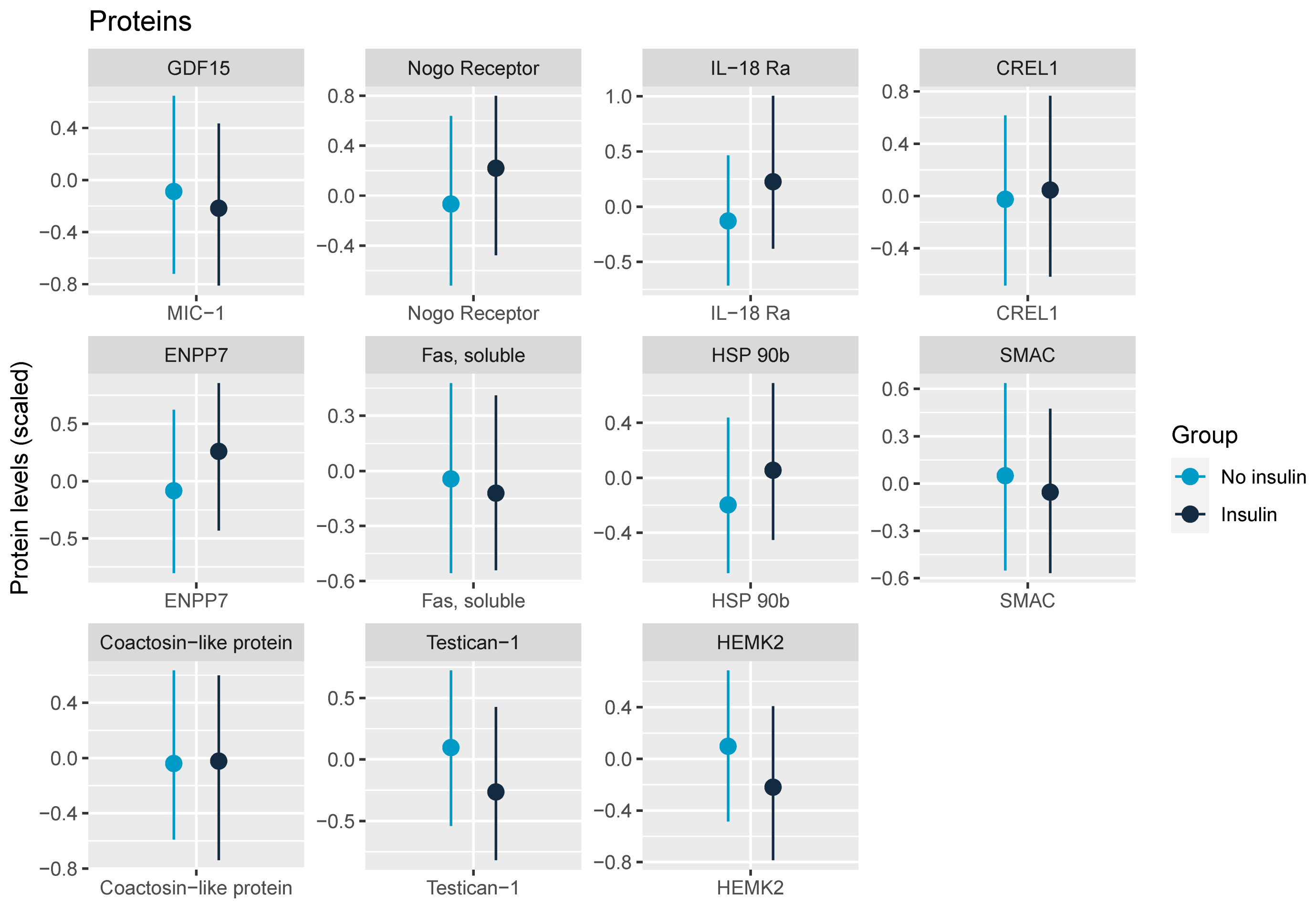

Supplemental Figure 6. Median scaled levels of the top proteins across the two discovery cohorts (DCS, GoDARTS). Light blue no incident insulin use, dark blue, incident insulin use. Data are presented as median with 25% and 75% quantile across the three cohorts.

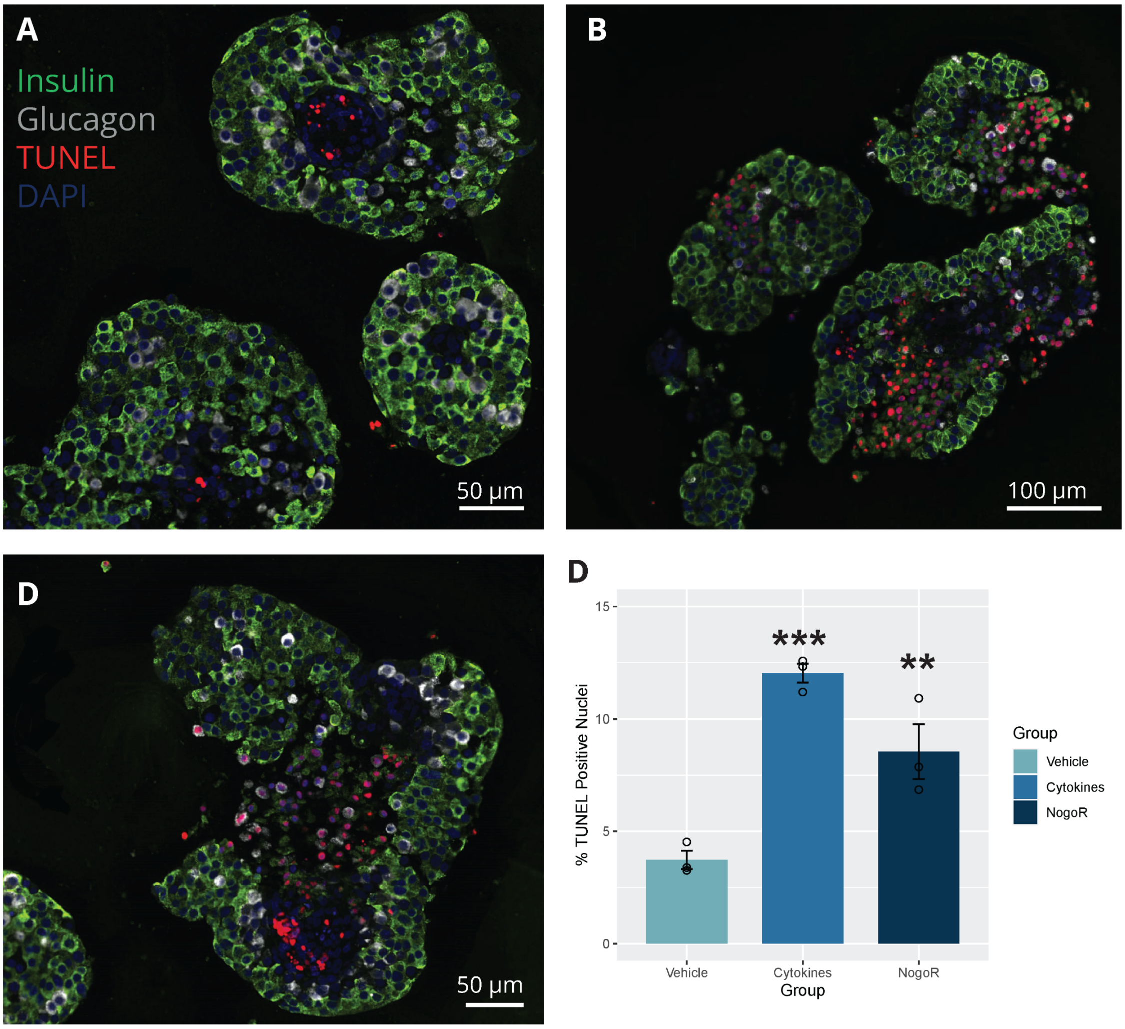

Supplemental Figure 7. The TUNEL staining of human pancreatic islets treated with cytokines and recombinant human NogoR protein. β-cell apoptosis was analyzed by staining of cultured human pancreatic islets of TUNEL, insulin, glucagon and DAPI. Representative islet images treated with vehicle (A), cytokines (B) and NogoR (C). (D) The percentage of TUNEL positive β-cells was calculated in islets from three (n=3) different human islet donors. Results shown are means ± SEM. **,***p=0.0085 and p=0.0005, respectively, *versus* the vehicle group by one-way ANOVA.

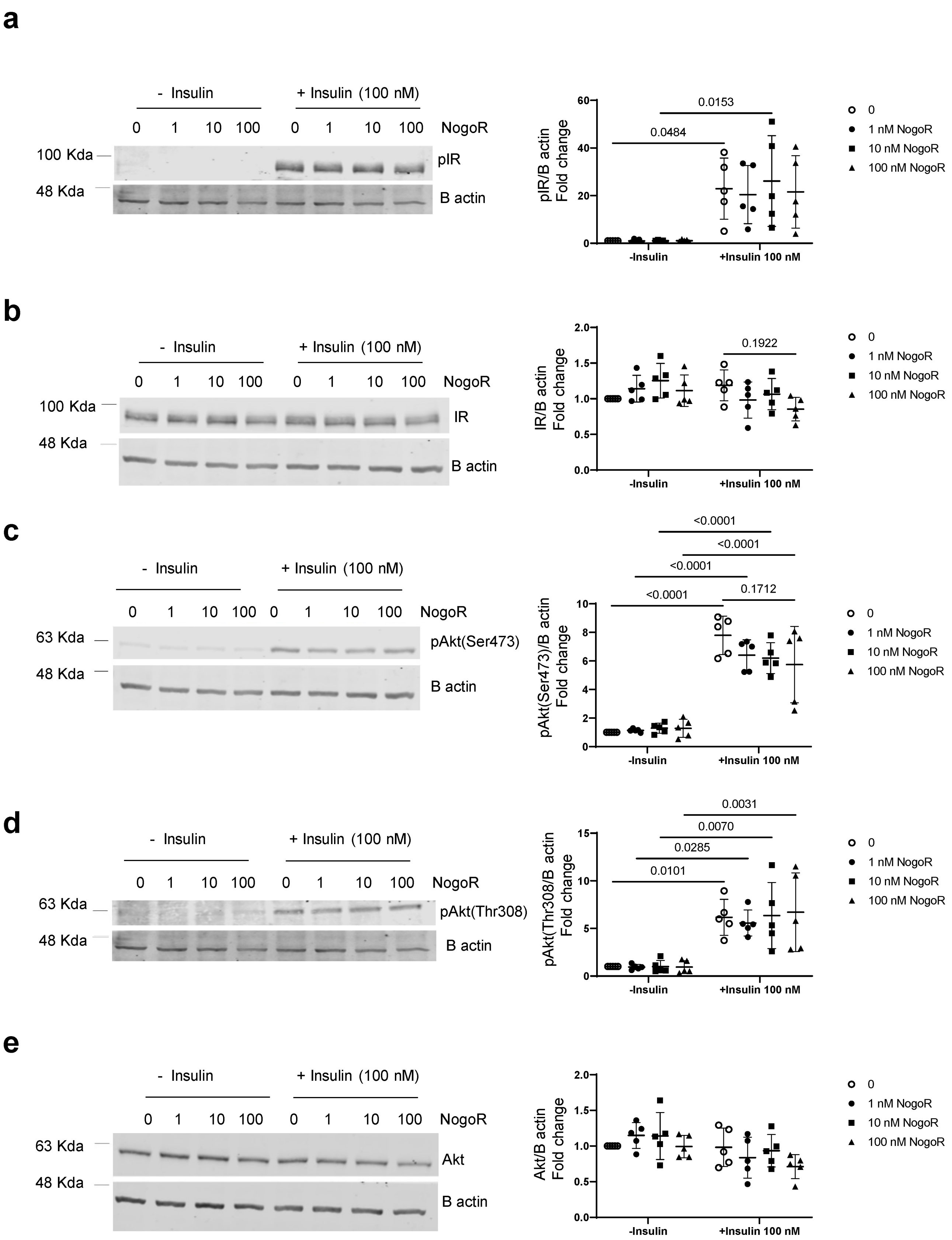
a
- Insulin
+ Insulin (100 nM)
0 1 10 100 0 1 10 100 NogoR
100 Kda
48 Kda
pIR
B actin
pIR/B actin
Fold change
0.0153
0.0484
-Insulin
+Insulin 100 nM
0
1 nM NogoR
10 nM NogoR
100 nM NogoR
b
- Insulin
+ Insulin (100 nM)
0 1 10 100 0 1 10 100 NogoR
100 Kda
48 Kda
IR
B actin
IR/B actin
Fold change
0.1922
-Insulin
+Insulin 100 nM
0
1 nM NogoR
10 nM NogoR
100 nM NogoR
c
- Insulin
+ Insulin (100 nM)
0 1 10 100 0 1 10 100 NogoR
63 Kda
48 Kda
pAkt(Ser473)
B actin
pAkt(Ser473)/B actin
Fold change
<0.0001
<0.0001
<0.0001
<0.0001
0.1712
-Insulin
+Insulin 100 nM
0
1 nM NogoR
10 nM NogoR
100 nM NogoR
d
- Insulin
+ Insulin (100 nM)
0 1 10 100 0 1 10 100 NogoR
63 Kda
48 Kda
pAkt(Thr308)
B actin
pAkt(Thr308/B actin
Fold change
0.0031
0.0070
0.0285
0.0101
-Insulin
+Insulin 100 nM
0
1 nM NogoR
10 nM NogoR
100 nM NogoR
e
- Insulin
+ Insulin (100 nM)
0 1 10 100 0 1 10 100 NogoR
63 Kda
48 Kda
Akt
B actin
Akt/B actin
Fold change
-Insulin
+Insulin 100 nM
0
1 nM NogoR
10 nM NogoR
100 nM NogoR

Supplementary Figure. 8. Effects of NogoR on insulin-stimulated phosphorylation of insulin receptor and Akt in primary mouse hepatocytes. Primary hepatocytes were plated in 12 well plates. They were serum and insulin starved for 24 hours and were then treated with different NogoR concentrations (1, 10, 100 nM) for 3 hrs prior to stimulation with 100 nM insulin for 15 min. Protein expression of Akt and its phosphorylated forms; pAkt (Ser473) and pAkt (Thr308), Insulin receptor beta (IR), and phospho Insulin receptor beta (pIR) were determined by western blot. B actin was used as loading control. The values were normalized to B actin and the fold change with respect to the untreated control was calculated for each condition. Two biologically independent experiments were performed. The first experiment was done using duplicate and the second experiment triplicate incubations. The replicates were compiled together (n=5 per condition) for statistical analysis by two-way ANOVA. P-values are indicated on the figure. Data are presented as mean ± SD.

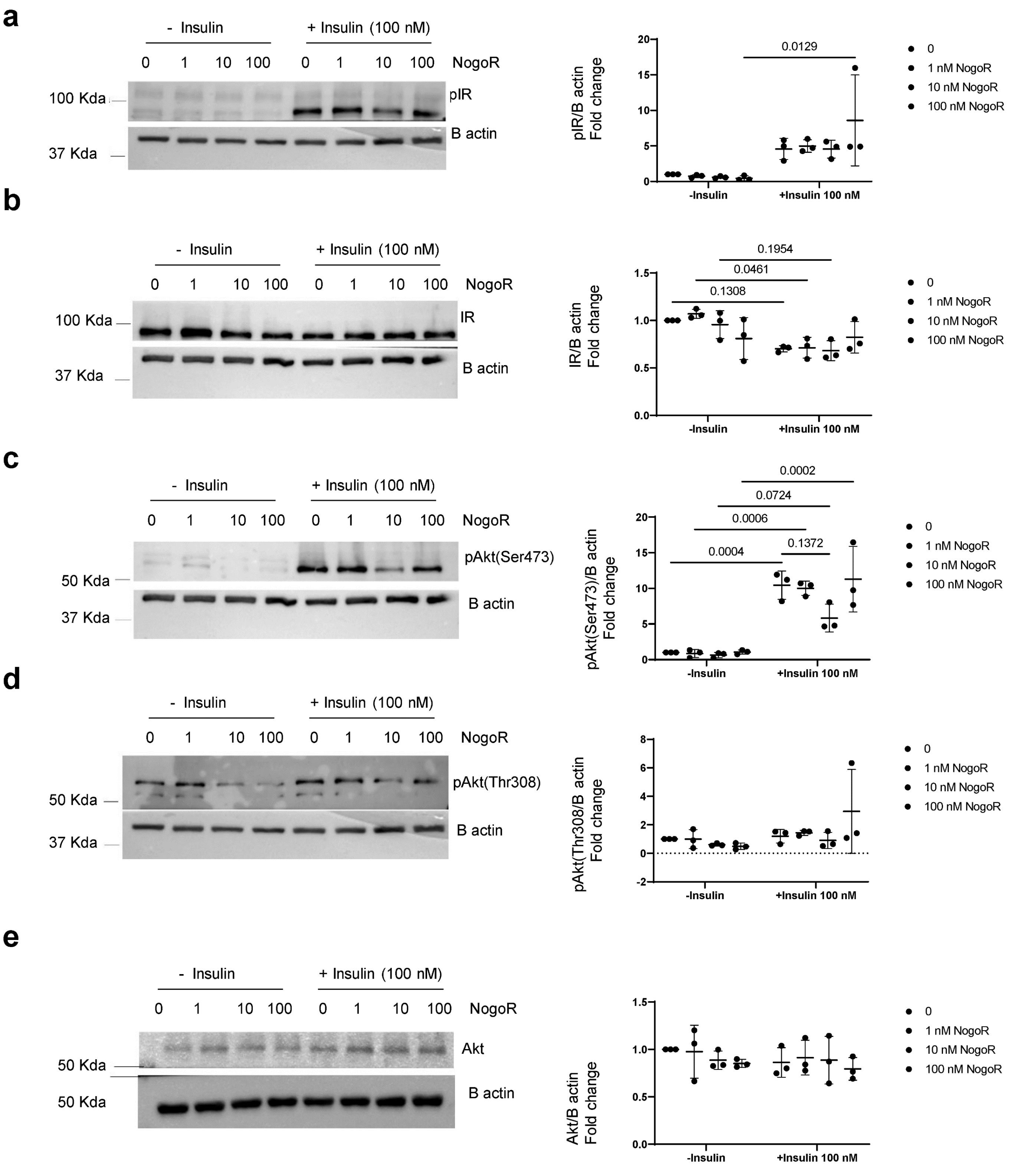
a
- Insulin
+ Insulin (100 nM)
0 1 10 100 0 1 10 100 NogoR
100 Kda
37 Kda
pIR
B actin
pIR/B actin
Fold change
0.0129
-Insulin
+Insulin 100 nM
0
1 nM NogoR
10 nM NogoR
100 nM NogoR
b
- Insulin
+ Insulin (100 nM)
0 1 10 100 0 1 10 100 NogoR
100 Kda
37 Kda
IR
B actin
IR/B actin
Fold change
0.1954
0.0461
0.1308
-Insulin
+Insulin 100 nM
0
1 nM NogoR
10 nM NogoR
100 nM NogoR
c
- Insulin
+ Insulin (100 nM)
0 1 10 100 0 1 10 100 NogoR
50 Kda
37 Kda
pAkt(Ser473)
B actin
pAkt(Ser473)/B actin
Fold change
0.0002
0.0724
0.0006
0.1372
0.0004
-Insulin
+Insulin 100 nM
0
1 nM NogoR
10 nM NogoR
100 nM NogoR
d
- Insulin
+ Insulin (100 nM)
0 1 10 100 0 1 10 100 NogoR
50 Kda
37 Kda
pAkt(Thr308)
B actin
pAkt(Thr308)/B actin
Fold change
-Insulin
+Insulin 100 nM
0
1 nM NogoR
10 nM NogoR
100 nM NogoR
e
- Insulin
+ Insulin (100 nM)
0 1 10 100 0 1 10 100 NogoR
50 Kda
50 Kda
Akt
B actin
Akt/B actin
Fold change
-Insulin
+Insulin 100 nM
0
1 nM NogoR
10 nM NogoR
100 nM NogoR

Supplementary Figure 9. Effects of NogoR on insulin-stimulated phosphorylation of Akt in adipocytes derived from C3H10T1/2 cells. C3H10T1/2 cells were plated in 12 well plates and differentiated to adipocytes. They were serum and insulin starved for 24 h and were then treated with different NogoR concentrations (1, 10, 100 nM) for 6 hrs prior to stimulation with 100 nM insulin for 15 min. Protein expression of Akt and its phosphorylated forms; pAkt (Ser473) and pAkt (Thr308), Insulin receptor beta (IR), and phospho Insulin receptor beta (pIR) were determined after SDS-PAGE and Western blotting. B actin was used as loading control. Values were normalized to B actin and the fold change with respect to the non-treated control was calculated for each condition. The results are from one biological experiment involving three separate incubations. Two-way ANOVA was used and P-values are indicated on the figure. Data are presented as mean ± SD.

a

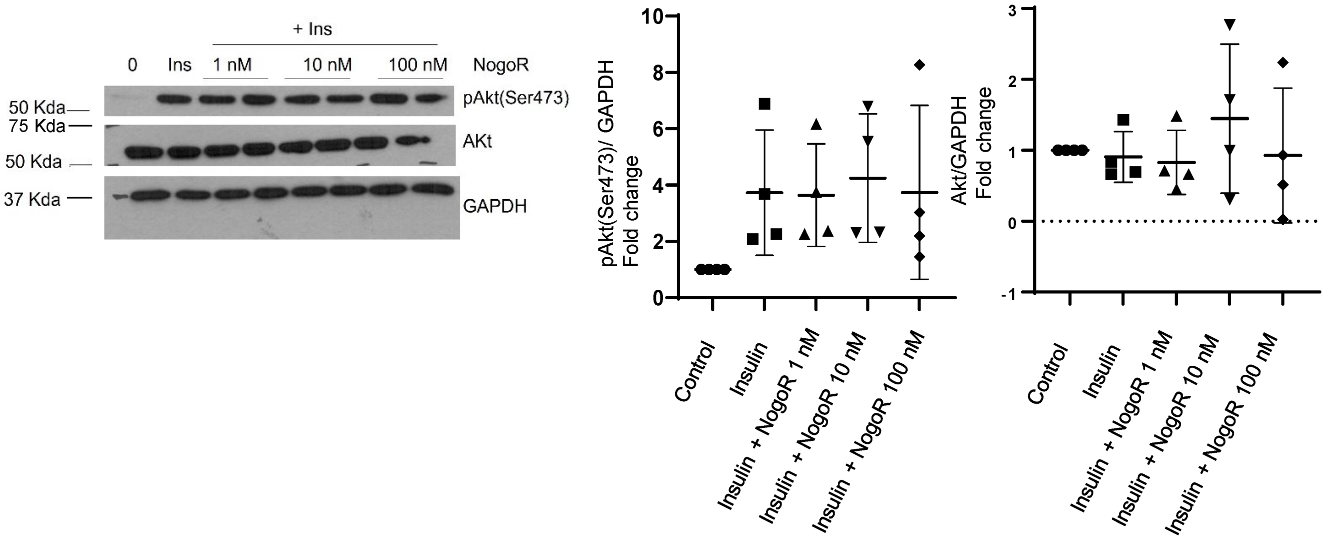

+ Ins
0
Ins
1 nM
10 nM
100 nM
NogoR
50 Kda
75 Kda
50 Kda
37 Kda
pAkt(Ser473)
AKt
GAPDH
pAkt(Ser473)/ GAPDH Fold change
Akt/GAPDH Fold change
Control
Insulin
Insulin + NogoR 1 nM
Insulin + NogoR 10 nM
Insulin + NogoR 100 nM

b

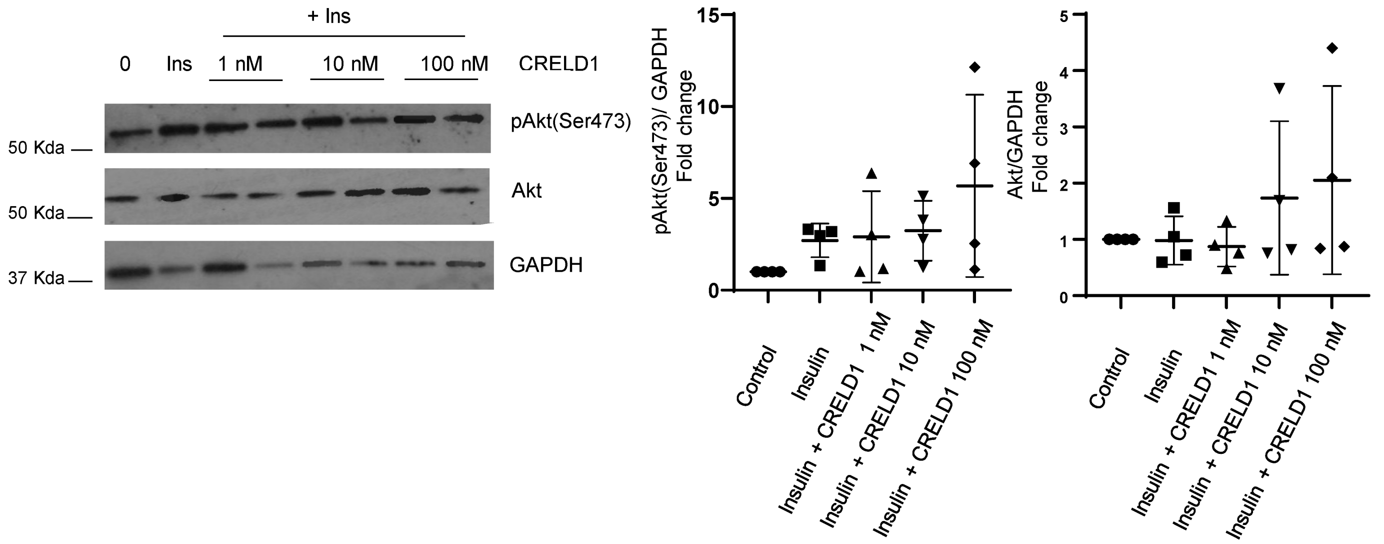

+ Ins
0
Ins
1 nM
10 nM
100 nM
CRELD1
50 Kda
50 Kda
37 Kda
pAkt(Ser473)
Akt
GAPDH
pAkt(Ser473)/ GAPDH Fold change
Akt/GAPDH Fold change
Control
Insulin
Insulin + CRELD1 1 nM
Insulin + CRELD1 10 nM
Insulin + CRELD1 100 nM

Supplementary Figure 10. Effects of NogoR and CRELD1 on insulin-stimulated phosphorylation of Akt in HepG2 cells. HepG2 cells were plated and then treated with different NogoR or CRELD1 concentrations (1, 10, 100 nM) for 3 h prior to stimulation with 100 nM insulin for 15 min. Protein levels of Akt and pAkt (Ser473) were determined by SDS-PAGE and Western blotting. GAPDH was used as the loading control. Values were normalized to GAPDH and the fold change with respect to the untreated control was calculated for each condition. The results are from four fully independent experiments (n=4), each involving incubations in duplicate. One way ANOVA with Tukeys' multiple comparison test or Kruskal Wallis test for multiple comparisons was used. None of the results were statistically significant. Data are presented as mean values +/- SD.

Table S1 Characteristics of the included discovery and validation cohorts

Metabolomics

	DCS	*GoDARTS (discovery)*	*GoDARTS (validation)*	*ANDIS (discovery)*	*ANDIS (validation)*
N	1267	897	699	811	1969
N events	227	311	307	74	361
%Males	55.96	56.97	56.22	60.54	59.9
Age (years)	63.83[57.37-70.77]	62.35[54.5-70.76]	64.61[56.78-72.67]	61.89[54.09-69.05]	62.76[55.53-70.05]
BMI (kg/m2)	30.34[26.7-33.1]	32.4[28-35.8]	31.54[27.45-34.6]	31.71[27.94-34.69]	30.83[27.08-33.92]
HbA1c (mmol/mol)	47.08[42-50]	55.54[46-61]	56.88[48-62]	60.06[46.0-68.0]	60.01[44.88-67.75]
C-peptide (nmol/L)	1.15[0.83-1.42]	2.1[1.35-2.71]	2.06[1.36-2.52]	1.32[0.93-1.6]	1.26[0.86-1.53]
HDL (mmol/L)	1.24[0.99-1.43]	1.31[1.06-1.5]	1.32[1.1-1.51]	1.2[0.96-1.4]	1.21[0.94-1.4]
LDL (mmol/L)	2.58[1.9-3.2]	2.15[1.58-2.63]	2.04[1.45-2.47]	3.21[2.5-3.9]	3.06[2.3-3.7]
Triglycerides (mmol/L)	1.79[1.15-2.18]	2.32[1.4-2.77]	2.31[1.46-2.76]	2.09[1.2-2.4]	2.07[1.2-2.4]
Diabetes duration (years)	2.63[1.43-3.76]	1.4[0.65-2.16]	3.95[3.36-4.54]	0[0-0]	0[0-0]
Metformin (%)	69.06	47.27	58.94	76.94	59.49
Sulfonylureas (%)	24.07	19.06	31.18	3.08	2.89

Lipidomics

	DCS	*GoDARTS (discovery)*	*ANDIS (discovery)*
N	900	899	809
N events	115	311	71
%Males	56.44	56.95	60.32
Age (years)	63.64[57.18-70.34]	62.32[54.47-70.75]	61.98[54.44-69.05]
BMI (kg/m2)	30.22[26.67-33.1]	32.42[28-35.8]	31.67[27.93-34.63]
HbA1c (mmol/mol)	47[41-49.73]	55.52[46-61]	60.01[46-68]
C-peptide (nmol/L)	1.16[0.84-1.42]	2.1[1.35-2.7]	1.32[0.93-1.6]
HDL (mmol/L)	1.24[1-1.44]	1.31[1.06-1.5]	1.21[0.96-1.4]
LDL (mmol/L)	2.61[1.9-3.2]	2.15[1.58-2.63]	3.21[2.5-3.9]
Triglycerides (mmol/L)	1.8[1.14-2.22]	2.29[1.4-2.75]	2.03[1.2-2.4]
Diabetes duration (years)	1.97[1.21-2.78]	1.4[0.65-2.16]	0[0-0]
Metformin (%)	66.78	47.27	76.88
Sulfonylureas (%)	20.56	19.13	3.09

Proteomics

	DCS	*GoDARTS*	*ANDIS (validation)*	*ACCELERATE (validation)*
N	589	599	1992	1850
N events	71	200	222	162
%Males	56.71	59.1	60.74	78.7
Age (years)	63.19[56.24-70.24]	61.9[54.09-70.22]	60.99[54.57-67.96]	67.17[61.4-72.5]
BMI (kg/m2)	30.25[26.7-33.1]	32.23[27.7-35.75]	31.93[28.2-34.9]	30.0[26.8-33.9]
HbA1c (mmol/mol)	46.84[41-49.73]	55.44[48-61]	59.44[45.91-66.71]	46.0[42.0-54.0]
C-peptide (nmol/L)	1.16[0.84-1.44]	2.15[1.37-2.76]	1.36[0.97-1.6]	3.44[2.52-4.59]
HDL (mmol/L)	1.23[1-1.4]	1.29[1.06-1.47]	1.18[0.94-1.4]	1.14[0.96-1.35]
LDL (mmol/L)	2.65[2-3.3]	2.19[1.62-2.69]	3.11[2.4-3.8]	1.99[1.63-2.43]
Triglycerides (mmol/L)	1.83[1.16-2.24]	2.32[1.43-2.77]	2.16[1.2-2.4]	1.53[1.13-2.06]
Diabetes duration (years)	1.45[1.1-2.06]	0.92[0.26-1.41]	0[0-0]	6.4[3.2-11.4]
Metformin (%)	67.23	45.41	71.03	68.5
Sulfonylureas (%)	18.68	17.20	3.16	31.6

Table S2 Cox proportional hazard ratio of base models without biomarkers.

	DCS		*GoDARTS*		*ANDIS*		*ACCELERATE*	
n	*3052*		*4679*		*6068*		1850	
n events	*536*		*1256*		*723*		161	
**Model 1 (age, sex, BMI)**								
Variable	*HR*	*P-value*	*HR*	*P-value*	*HR*	*P-value*	*HR*	*P-value*
Age	0.97[0.96-0.98]	$1.64 \cdot 10^{-15}$	0.96[0.96-0.97]	$8.81 \cdot 10^{-30}$	0.99[0.99-1.01]	0.56	0.98[0.96-0.99]	0.01
Sex	1.09[0.91-1.31]	0.38	0.93[0.82-1.04]	0.19	1.05[0.90-1.23]	0.54	1.10[0.75-1.61]	0.62
BMI	1.00[0.98-1.02]	0.48	1.01[1.00-1.02]	0.01	0.97[0.96-0.99]	0.0002	1.01[0.99-1.04]	0.24
**Model 2 (M1 + HDL, C-peptide)**								
Variable	*DCS*		*GoDARTS*		*ANDIS*		*ACCELERATE*	
	HR	*P-value*	*HR*	*P-value*	*HR*	*P-value*	*HR*	*P-value*
Age	0.98[0.97-0.99]	$3.04 \cdot 10^{-5}$	0.97[0.96-0.98]	$4.37 \cdot 10^{-26}$	1.00[0.99-1.01]	0.509	0.97[0.96-0.99]	0.01
Sex	0.83[0.68-1.02]	0.08	0.98[0.87-1.11]	0.76	1.14[0.97-1.34]	0.10	1.13[0.77-1.67]	0.53
BMI	1.02[1.00-1.04]	0.02	1.01[1.00-1.02]	0.06	0.99[0.97-1.00]	0.10	1.00[0.98-1.03]	0.86
HDL	0.27[0.18-0.40]	$3.20 \cdot 10^{-11}$	0.67[0.55-0.82]	$8.28 \cdot 10^{-5}$	0.64[0.50-0.81]	0.0003	0.78[0.43-1.43]	0.43
C-peptide	0.35[0.30-0.40]	$1.35 \cdot 10^{-46}$	1.02[0.90-1.16]	0.76	0.61[0.50-0.74]	$1.48 \cdot 10^{-6}$	1.37[0.92-2.04]	0.12
**Model 3 (M2 + diabetes duration + glucose-lowering drugs)**								
Variable	*DCS*		*GoDARTS*		*ANDIS*		*ACCELERATE*	
	HR	*P-value*	*HR*	*P-value*	*HR*	*P-value*	*HR*	*P-value*
Age	0.98[0.97-0.99]	0.0002	0.97[0.96-0.97]	$1.30 \cdot 10^{-27}$	1.00[0.99-1.01]	0.63	0.96[0.94-0.98]	$9.40 \cdot 10^{-5}$
Sex	0.81[0.66-1]	0.05	0.99[0.88-1.12]	0.934	1.13[0.97-1.33]	0.13	1.03[0.70-1.53]	0.88
BMI	1.02[1-1.04]	0.02	1.01[0.99-1.02]	0.11	0.99[0.97-1.01]	0.13	1.01[0.98-1.04]	0.57
HDL	0.29[0.2-0.43]	$2.64 \cdot 10^{-10}$	0.59[0.49-0.72]	$2.72 \cdot 10^{-7}$	0.70[0.55-0.89]	0.0035	0.80[0.44-1.45]	0.46
C-peptide	0.36[0.31-0.42]	$1.46 \cdot 10^{-41}$	0.96[0.85-1.09]	0.55	0.67[0.55-0.82]	$8.71 \cdot 10^{-5}$	1.50[1.02-2.22]	0.04
Diabetes duration	1.00[1.00-1.00]	0.39	1.06[1.03-1.08]	$1.51 \cdot 10^{-6}$	0.84[0.61-1.17]	0.31	1.05[1.03-1.07]	$6.02 \cdot 10^{-6}$
Glucose lowering drugs	3.42[2.63-4.45]	$5.46 \cdot 10^{-20}$	2.33[1.99-2.73]	$1.22 \cdot 10^{-25}$	0.52[0.45-0.61]	$5.81 \cdot 10^{-16}$	4.73[1.50-14.92]	$8.09 \cdot 10^{-3}$

M1, model 1; M2, model 2; HR, hazard ratio. Numbers between brackets represent confidence intervals. Statistical test: Cox proportional hazard model.

Table S3 Cox proportional hazard models for the metabolites

	**Discovery**																
	**Model 1 (age, sex, BMI)**									**Model 2 (M1 + HDL, C-peptide)**				**Model 3 (M2 + diabetes duration, glucose-lowering drugs)**			
var	*HR*	*Lower*	*Upper*	*Adj.lower*	*Adj.upper*	*P-value*	*I2*	*Het*	*FDR*	*HR*	*Lower*	*Upper*	*P-value*	*HR*	*Lower*	*Upper*	*P-value*
AADA	1.11	1.04	1.19	1.01	1.22	1.77E-03	0.00	0.34	0.03	1.11	1.04	1.19	3.11E-03	1.06	0.95	1.18	0.30
Hcit	1.12	1.04	1.21	1.00	1.25	3.96E-03	0.00	0.31	0.04	1.12	1.03	1.20	4.42E-03	0.70	0.42	1.15	0.16
GCA	1.09	1.01	1.17	0.98	1.20	0.02	0.00	0.30	0.10	1.09	1.01	1.17	0.02	1.07	0.89	1.30	0.47
TCA	1.06	1.01	1.12	0.99	1.15	0.02	0.00	0.23	0.10	1.06	1.01	1.12	0.02	0.82	0.30	2.21	0.69
Ile	1.09	1.00	1.20	0.96	1.25	0.05	0.00	0.32	0.19	1.07	0.98	1.18	0.13	1.08	0.76	1.53	0.69
Cit	0.93	0.85	1.01	0.95	1.21	0.10	0.00	0.23	0.28	0.95	0.86	1.04	0.22	1.15	0.97	1.35	0.10
Leu	1.07	0.99	1.17	0.82	1.05	0.10	0.00	0.39	0.28	1.06	0.97	1.15	0.19	0.88	0.77	1.01	0.08
IndS	1.11	0.96	1.30	0.90	1.38	0.17	0.68	0.01	0.39	1.08	0.94	1.24	0.29	1.17	1.02	1.36	0.03
Kynu	1.04	0.95	1.13	0.92	1.17	0.39	0.00	0.19	0.78	1.04	0.95	1.13	0.41	0.87	0.73	1.05	0.14
Ala	1.04	0.94	1.16	0.90	1.20	0.41	0.21	0.11	0.78	1.04	0.94	1.15	0.46	0.96	0.63	1.45	0.83
Phe	1.03	0.95	1.13	0.92	1.16	0.46	0.04	0.15	0.78	1.02	0.94	1.11	0.59	1.04	0.57	1.88	0.90
Trp	0.97	0.90	1.05	0.87	1.09	0.49	0.00	0.48	0.78	0.98	0.90	1.06	0.63	0.82	0.69	0.97	0.02
Tyr	0.98	0.90	1.07	0.87	1.11	0.67	0.00	0.68	0.85	0.99	0.91	1.08	0.81	0.98	0.89	1.07	0.60
Gln	0.98	0.90	1.07	0.85	1.14	0.70	0.01	0.15	0.85	0.97	0.88	1.07	0.57	1.33	0.74	2.38	0.34
GUDCA	1.01	0.93	1.11	0.78	1.23	0.79	0.00	0.19	0.85	1.02	0.94	1.12	0.63	1.07	0.97	1.19	0.18
Taurine	0.99	0.91	1.07	0.89	1.15	0.80	0.00	0.17	0.85	0.99	0.91	1.07	0.75	0.97	0.55	1.72	0.93
Gly	0.98	0.84	1.14	0.82	1.18	0.80	0.59	0.03	0.85	0.98	0.84	1.14	0.80	0.97	0.79	1.19	0.75
Glu	0.98	0.87	1.12	0.87	1.12	0.81	0.53	0.04	0.85	0.97	0.84	1.12	0.64	0.95	0.67	1.34	0.76
SDMA.ADMA	1.00	0.90	1.12	0.85	1.18	0.98	0.29	0.09	0.98	1.01	0.90	1.14	0.84	0.98	0.84	1.14	0.77

Statistical test: Cox proportional hazard models.

Table S4. pQTLs associated with top aptamers.

Seq_Id	*ID*	*Protein*	*Cis-pQTL*	*P-value*	*Source*
4374-45_ 2	SL003869	GDF15/MIC-1	rs1058587	3.8·10-477	Ferkingstad
5105-2_3	SL005208	Nogo receptor	rs75766	4.1·10-204	Ferkingstad
3446-7_2	SL004152	IL18 Ra	rs1420106	1.1·10-273	Sun
7628-40_3	SL012774	CRELD1	rs4234585	1.3·10-2640	Ferkingstad
4435-66_2	SL009045	ENPP7	rs11871061	7.9·10-2953	Ferkingstad
5392-73_2	SL002731	Fas, soluble	rs7911226	1.0·10-152	Ferkingstad
5467-15_3	SL000454	HSP-90B	rs190077456	7.80·10-6	Ferkingstad
3122-6_2	SL003733	SMAC	rs111553874	1.10·10-4	Ferkingstad
4905-63_1	SL004814	Coactosin−like protein	rs173777	3.27·10-4	Ferkingstad
5490-53_3	SL010384	Testican-1	rs11744278	7.5·10-4	Ferkingstad
11096-57_3	SL018921	HEMK2	rs1470967471	0.003155	Ferkingstad

Only SNPs included in the table with MAF ≥ 1% and 1Mb from transcription start or end site. The study from Ferkingstad et al is a GWAS based on 35,559 individuals

Lipidomics Minimal Reporting Checklist

General Lipidomics Workflow

INTERNATIONAL LIPIDOMICS SOCIETY

Overall study design

Title of the study	Novel biomarkers for glycaemic deterioration in type 2 diabetes: an IMI-RHAPSODY study		
Principle investigator	Prof Guy Rutter		
Institution	Imperial College London		
Corresponding Email	g.rutter@imperial.ac.uk		
Document creation date	02/16/2023	Clinical	No
Is the workflow targeted or untargeted?	Untargeted		

Lipid extraction

Extraction method	2-phase system	2-phase system	MTBE
pH adjustment	None	Were internal standards added prior extraction?	Yes

Analytical platform

MS type	Orbitrap	Resolution at m/z 200 at MS1	280000
MS vendor	Thermo	Mass accuracy in ppm at MS1	1
Ion source	ESI	Mass window for precursor ion isolation (in Da total isolation window)	1
Direct type	Chip	Mass resolution for detected ion at MS2	High resolution
MS Level	MS1, MS2	Resolution at m/z 200 at MS2	17500
Mass resolution for detected ion at MS1	High resolution	Mass accuracy in ppm at MS2	3

Quality control

Blanks	Yes	Quality control	Yes
Type of Blanks	Extraction blank	Type of QC sample	Reference material

Method qualification and validation

Method validation	Yes	Precison	Yes
Lipid recovery	Yes	Accuracy	No
Dynamic quantification range	Yes	Guidelines followed	None
Limit of quantitation (LOQ)/Limit of detection (LOD)	Yes		

Reporting

Are reported raw data uploaded into repository?	Available on request	Raw data upload	No

Sample Descriptions

Plasma samples / Human / Plasma

Temperature handling original sample	4-8 °C	Were samples stored under inert gas?	No
Instant sample preparation	No	Additional preservation methods	No
Storage temperature	-80 °C	Biobank samples	Yes
Additives	None		

Lipid Class Descriptions

Lipid class LPC[M+CH3COO]- / Lipid identification

Lipid class	LPC	MS1 verified by standard	Yes
MS Level	MS1	Background check at MS1	Yes
Identification level	Species level	Check isomer overlap	Yes
Polarity mode	Negative	Lipid Identification Software	LipotypeXplorer
Type of negative (precursor)ion	[M+CH3COO]-	Data manipulation	Centroiding
Isotope correction at MS1	Type 2	Nomenclature for intact lipid molecule	Yes

Lipid class LPC[M+CH3COO]- / For additional separation methods/analytical dimension

Quantitative	Yes	Limit of quantification	S/N ratio
Internal lipid standard(s)	LPC 12:0	Normalization to reference	No
Type of quantification	Internal standard amount	Lipid Quantification Software	LipotypeXplorer
Response correction	No	Batch correction	Normalization by reference material/QC
Type I isotope correction	Yes		

Lipid class Cer[M+CH3COO]- / Lipid identification

Lipid class	Cer	MS1 verified by standard	Yes
MS Level	MS1	Background check at MS1	Yes
Identification level	Species level	Check isomer overlap	Yes
Polarity mode	Negative	Lipid Identification Software	LipotypeXplorer
Type of negative (precursor)ion	[M+CH3COO]-	Data manipulation	Centroiding
Isotope correction at MS1	Type 2	Nomenclature for intact lipid molecule	Yes

Lipid class Cer[M+CH3COO]- / For additional separation methods/analytical dimension

Quantitative	Yes	Limit of quantification	S/N ratio
Internal lipid standard(s)	Cer 35:1;O2	Normalization to reference	No
Type of quantification	Internal standard amount	Lipid Quantification Software	LipotypeXplorer
Response correction	No	Batch correction	Normalization by reference material/QC
Type I isotope correction	Yes		

Lipid class SM[M+CH3COO]- / Lipid identification

Lipid class	SM	MS1 verified by standard	Yes
MS Level	MS1	Background check at MS1	Yes
Identification level	Species level	Check isomer overlap	Yes
Polarity mode	Negative	Lipid Identification Software	LipotypeXplorer
Type of negative (precursor)ion	[M+CH3COO]-	Data manipulation	Centroiding
Isotope correction at MS1	Type 2	Nomenclature for intact lipid molecule	Yes

Lipid class SM[M+CH3COO]- / For additional separation methods/analytical dimension

Quantitative	Yes	Limit of quantification	S/N ratio
Internal lipid standard(s)	SM 30:1;O2	Normalization to reference	No
Type of quantification	Internal standard amount	Lipid Quantification Software	LipotypeXplorer
Response correction	No	Batch correction	Normalization by reference material/QC
Type I isotope correction	Yes		

Lipid class ST 27:1;1[M+NH4]+ / Lipid identification

Lipid class	ST 27:1;1	MS1 verified by standard	Yes
MS Level	MS1	Background check at MS1	Yes
Identification level	Species level	Check isomer overlap	Yes
Polarity mode	Positive	Lipid Identification Software	LipotypeXplorer
Type of positive (precursor)ion	[M+NH4]+	Data manipulation	Centroiding
Isotope correction at MS1	Type 2	Nomenclature for intact lipid molecule	Yes

Lipid class ST 27:1;1[M+NH4]+ / For additional separation methods/analytical dimension

Quantitative	Yes	Limit of quantification	S/N ratio
Internal lipid standard(s)	Chol-D6	Normalization to reference	No
Type of quantification	Internal standard amount	Lipid Quantification Software	LipotypeXplorer
Response correction	No	Batch correction	Normalization by reference material/QC
Type I isotope correction	Yes		

Lipid class PC O-a[M+CH3COO]- / Lipid identification

Lipid class	PC O-a	MS1 verified by standard	Yes
MS Level	MS1, MS2	MS2 verified by standard	Yes
Identification level	Molecular species level	Background check at MS1	Yes
Polarity mode	Negative	Background check at MS2	Yes
Type of negative (precursor)ion	[M+CH3COO]-	Check isomer overlap	Yes
How many fragments used for ID	2 fragments	Lipid Identification Software	LipotypeXplorer
Fragment ion 1	fatty acyl ion	Data manipulation	Centroiding
Fragment ion 2	fatty acyl neutral loss	Nomenclature for intact lipid molecule	Yes
Isotope correction at MS1	Type 2	Nomenclature for fragment ions	Yes
Isotope correction at MS2	Type 2		

Lipid class PC O-a[M+CH3COO]- / For additional separation methods/analytical dimension

Quantitative	Yes	Limit of quantification	S/N ratio
Internal lipid standard(s)	PC 17:0/17:0	Normalization to reference	No
Type of quantification	Internal standard amount	Lipid Quantification Software	LipotypeXplorer
Response correction	No	Batch correction	Normalization by reference material/QC
Type I isotope correction	Yes		

Lipid class PC O-p[M+CH3COO]- / Lipid identification

Lipid class	PC O-p	MS1 verified by standard	Yes
MS Level	MS1, MS2	MS2 verified by standard	Yes
Identification level	Molecular species level	Background check at MS1	Yes
Polarity mode	Negative	Background check at MS2	Yes
Type of negative (precursor)ion	[M+CH3COO]-	Check isomer overlap	Yes
How many fragments used for ID	2 fragments	Lipid Identification Software	LipotypeXplorer
Fragment ion 1	fatty acyl ion	Data manipulation	Centroiding
Fragment ion 2	fatty acyl neutral loss	Nomenclature for intact lipid molecule	Yes
Isotope correction at MS1	Type 2	Nomenclature for fragment ions	Yes
Isotope correction at MS2	Type 2		

Lipid class PC O-p[M+CH3COO]- / For additional separation methods/analytical dimension

Quantitative	Yes	Limit of quantification	S/N ratio
Internal lipid standard(s)	PC 17:0/17:0	Normalization to reference	No
Type of quantification	Internal standard amount	Lipid Quantification Software	LipotypeXplorer
Response correction	No	Batch correction	Normalization by reference material/QC
Type I isotope correction	Yes		

Lipid class PE[M-H]- / Lipid identification

Lipid class	PE	MS1 verified by standard	Yes
MS Level	MS1, MS2	MS2 verified by standard	Yes
Identification level	Molecular species level	Background check at MS1	Yes
Polarity mode	Negative	Background check at MS2	Yes
Type of negative (precursor)ion	[M-H]-	Check isomer overlap	Yes
How many fragments used for ID	2 fragments	Lipid Identification Software	LipotypeXplorer
Fragment ion 1	fatty acyl ion	Data manipulation	Centroiding
Fragment ion 2	fatty acyl ion	Nomenclature for intact lipid molecule	Yes
Isotope correction at MS1	Type 2	Nomenclature for fragment ions	Yes
Isotope correction at MS2	Type 2		

Lipid class PE[M-H]- / For additional separation methods/analytical dimension

Quantitative	Yes	Limit of quantification	S/N ratio
Internal lipid standard(s)	PE 17:0/17:0	Normalization to reference	No
Type of quantification	Internal standard amount	Lipid Quantification Software	LipotypeXplorer
Response correction	No	Batch correction	Normalization by reference material/QC
Type I isotope correction	Yes		

Lipid class PE O-a[M-H]- / Lipid identification

Lipid class	PE O-a	MS1 verified by standard	Yes
MS Level	MS1, MS2	MS2 verified by standard	Yes
Identification level	Molecular species level	Background check at MS1	Yes
Polarity mode	Negative	Background check at MS2	Yes
Type of negative (precursor)ion	[M-H]-	Check isomer overlap	Yes
How many fragments used for ID	2 fragments	Lipid Identification Software	LipotypeXplorer
Fragment ion 1	fatty acyl ion	Data manipulation	Centroiding
Fragment ion 2	fatty acyl neutral loss	Nomenclature for intact lipid molecule	Yes
Isotope correction at MS1	Type 2	Nomenclature for fragment ions	Yes
Isotope correction at MS2	Type 2		

Lipid class PE O-a[M-H]- / For additional separation methods/analytical dimension

Quantitative	Yes	Limit of quantification	S/N ratio
Internal lipid standard(s)	PE 17:0/17:0	Normalization to reference	No
Type of quantification	Internal standard amount	Lipid Quantification Software	LipotypeXplorer
Response correction	No	Batch correction	Normalization by reference material/QC
Type I isotope correction	Yes		

Lipid class PE O-p[M-H]- / Lipid identification

Lipid class	PE O-p	MS1 verified by standard	Yes
MS Level	MS1, MS2	MS2 verified by standard	Yes
Identification level	Molecular species level	Background check at MS1	Yes
Polarity mode	Negative	Background check at MS2	Yes
Type of negative (precursor)ion	[M-H]-	Check isomer overlap	Yes
How many fragments used for ID	2 fragments	Lipid Identification Software	LipotypeXplorer
Fragment ion 1	fatty acyl ion	Data manipulation	Centroiding
Fragment ion 2	fatty acyl neutral loss	Nomenclature for intact lipid molecule	Yes
Isotope correction at MS1	Type 2	Nomenclature for fragment ions	Yes
Isotope correction at MS2	Type 2		

Lipid class PE O-p[M-H]- / For additional separation methods/analytical dimension

Quantitative	Yes	Limit of quantification	S/N ratio
Internal lipid standard(s)	PE 17:0/17:0	Normalization to reference	No
Type of quantification	Internal standard amount	Lipid Quantification Software	LipotypeXplorer
Response correction	No	Batch correction	Normalization by reference material/QC
Type I isotope correction	Yes		

Lipid class PI[M-H]- / Lipid identification

Lipid class	PI	MS1 verified by standard	Yes
MS Level	MS1, MS2	MS2 verified by standard	Yes
Identification level	Molecular species level	Background check at MS1	Yes
Polarity mode	Negative	Background check at MS2	Yes
Type of negative (precursor)ion	[M-H]-	Check isomer overlap	Yes
How many fragments used for ID	2 fragments	Lipid Identification Software	LipotypeXplorer
Fragment ion 1	fatty acyl ion	Data manipulation	Centroiding
Fragment ion 2	fatty acyl ion	Nomenclature for intact lipid molecule	Yes
Isotope correction at MS1	Type 2	Nomenclature for fragment ions	Yes
Isotope correction at MS2	Type 2		

Lipid class PI[M-H]- / For additional separation methods/analytical dimension

Quantitative	Yes	Limit of quantification	S/N ratio
Internal lipid standard(s)	PI 16:0/16:0	Normalization to reference	No
Type of quantification	Internal standard amount	Lipid Quantification Software	LipotypeXplorer
Response correction	No	Batch correction	Normalization by reference material/QC
Type I isotope correction	Yes		

Lipid class SE 27:1[M+NH4]+ / Lipid identification

Lipid class	SE 27:1	MS1 verified by standard	Yes
MS Level	MS1, MS2	MS2 verified by standard	Yes
Identification level	Molecular species level	Background check at MS1	Yes
Polarity mode	Positive	Background check at MS2	Yes
Type of positive (precursor)ion	[M+NH4]+	Check isomer overlap	Yes
How many fragments used for ID	1 fragment	Lipid Identification Software	LipotypeXplorer
Fragment ion 1	fatty acyl neutral loss	Data manipulation	Centroiding
Isotope correction at MS1	Type 2	Nomenclature for intact lipid molecule	Yes
Isotope correction at MS2	Type 2	Nomenclature for fragment ions	Yes

Lipid class SE 27:1[M+NH4]+ / For additional separation methods/analytical dimension

Quantitative	Yes	Limit of quantification	S/N ratio
Internal lipid standard(s)	CE 20:0	Normalization to reference	No
Type of quantification	Internal standard amount	Lipid Quantification Software	LipotypeXplorer
Response correction	No	Batch correction	Normalization by reference material/QC
Type I isotope correction	Yes		

Lipid class DG[M+NH4]+ / Lipid identification

Lipid class	DG	MS1 verified by standard	Yes
MS Level	MS1, MS2	MS2 verified by standard	Yes
Identification level	Molecular species level	Background check at MS1	Yes
Polarity mode	Positive	Background check at MS2	Yes
Type of positive (precursor)ion	[M+NH4]+	Check isomer overlap	Yes
How many fragments used for ID	2 fragments	Lipid Identification Software	LipotypeXplorer
Fragment ion 1	fatty acyl neutral loss	Data manipulation	Centroiding
Fragment ion 2	fatty acyl neutral loss	Nomenclature for intact lipid molecule	Yes
Isotope correction at MS1	Type 2	Nomenclature for fragment ions	Yes
Isotope correction at MS2	Type 2		

Lipid class DG[M+NH4]+ / For additional separation methods/analytical dimension

Quantitative	Yes	Limit of quantification	S/N ratio
Internal lipid standard(s)	DAG 17:0/17:0	Normalization to reference	No
Type of quantification	Internal standard amount	Lipid Quantification Software	LipotypeXplorer
Response correction	No	Batch correction	Normalization by reference material/QC
Type I isotope correction	Yes		

Lipid class TG[M+NH4]+ / Lipid identification

Lipid class	TG	Isotope correction at MS2	Type 2
MS Level	MS1, MS2	MS1 verified by standard	Yes
Identification level	Molecular species level	MS2 verified by standard	Yes
Polarity mode	Positive	Background check at MS1	Yes
Type of positive (precursor)ion	[M+NH4]+	Background check at MS2	Yes
How many fragments used for ID	3 fragments	Check isomer overlap	Yes
Fragment ion 1	fatty acyl neutral loss	Lipid Identification Software	LipotypeXplorer
Fragment ion 2	fatty acyl neutral loss	Data manipulation	Centroiding
Fragment ion 3	fatty acyl neutral loss	Nomenclature for intact lipid molecule	Yes
Isotope correction at MS1	Type 2	Nomenclature for fragment ions	Yes

Lipid class TG[M+NH4]+ / For additional separation methods/analytical dimension

Quantitative	Yes	Limit of quantification	S/N ratio
Internal lipid standard(s)	TAG 17:0/17:0/17:0	Normalization to reference	No
Type of quantification	Internal standard amount	Lipid Quantification Software	LipotypeXplorer
Response correction	No	Batch correction	Normalization by reference material/QC
Type I isotope correction	Yes		

Lipid class HexCer[M+CH3COO]- / Lipid identification

Lipid class	HexCer	MS1 verified by standard	Yes
MS Level	MS1	Background check at MS1	Yes
Identification level	Species level	Check isomer overlap	Yes
Polarity mode	Negative	Lipid Identification Software	LipotypeXplorer
Type of negative (precursor)ion	[M+CH3COO]-	Data manipulation	Centroiding
Isotope correction at MS1	Type 2	Nomenclature for intact lipid molecule	Yes

Lipid class HexCer[M+CH3COO]- / For additional separation methods/analytical dimension

Quantitative	Yes	Limit of quantification	S/N ratio
Internal lipid standard(s)	HexCer 30:1;O2	Normalization to reference	No
Type of quantification	Internal standard amount	Lipid Quantification Software	LipotypeXplorer
Response correction	No	Batch correction	Normalization by reference material/QC
Type I isotope correction	Yes		

Lipid class LPC O-a[M+CH3COO]- / Lipid identification

Lipid class	LPC O-a	MS1 verified by standard	Yes
MS Level	MS1	Background check at MS1	Yes
Identification level	Species level	Check isomer overlap	Yes
Polarity mode	Negative	Lipid Identification Software	LipotypeXplorer
Type of negative (precursor)ion	[M+CH3COO]-	Data manipulation	Centroiding
Isotope correction at MS1	Type 2	Nomenclature for intact lipid molecule	Yes

Lipid class LPC O-a[M+CH3COO]- / For additional separation methods/analytical dimension

Quantitative	Yes	Limit of quantification	S/N ratio
Internal lipid standard(s)	LPC 12:0	Normalization to reference	No
Type of quantification	Internal standard amount	Lipid Quantification Software	LipotypeXplorer
Response correction	No	Batch correction	Normalization by reference material/QC
Type I isotope correction	Yes		

Lipid class LPC O-p[M+CH3COO]- / Lipid identification

Lipid class	LPC O-p	MS1 verified by standard	Yes
MS Level	MS1	Background check at MS1	Yes
Identification level	Species level	Check isomer overlap	Yes
Polarity mode	Negative	Lipid Identification Software	LipotypeXplorer
Type of negative (precursor)ion	[M+CH3COO]-	Data manipulation	Centroiding
Isotope correction at MS1	Type 2	Nomenclature for intact lipid molecule	Yes

Lipid class LPC O-p[M+CH3COO]- / For additional separation methods/analytical dimension

Quantitative	Yes	Limit of quantification	S/N ratio
Internal lipid standard(s)	LPC 12:0	Normalization to reference	No
Type of quantification	Internal standard amount	Lipid Quantification Software	LipotypeXplorer
Response correction	No	Batch correction	Normalization by reference material/QC
Type I isotope correction	Yes		

Lipid class LPE O-a[M-H]- / Lipid identification

Lipid class	LPE O-a	MS1 verified by standard	Yes
MS Level	MS1	Background check at MS1	Yes
Identification level	Species level	Check isomer overlap	Yes
Polarity mode	Negative	Lipid Identification Software	LipotypeXplorer
Type of negative (precursor)ion	[M-H]-	Data manipulation	Centroiding
Isotope correction at MS1	Type 2	Nomenclature for intact lipid molecule	Yes

Lipid class LPE O-a[M-H]- / For additional separation methods/analytical dimension

Quantitative	Yes	Limit of quantification	S/N ratio
Internal lipid standard(s)	LPE 17:1	Normalization to reference	No
Type of quantification	Internal standard amount	Lipid Quantification Software	LipotypeXplorer
Response correction	No	Batch correction	Normalization by reference material/QC
Type I isotope correction	Yes		

Lipid class LPE O-p[M-H]- / Lipid identification

Lipid class	LPE O-p	MS1 verified by standard	Yes
MS Level	MS1	Background check at MS1	Yes
Identification level	Species level	Check isomer overlap	Yes
Polarity mode	Negative	Lipid Identification Software	LipotypeXplorer
Type of negative (precursor)ion	[M-H]-	Data manipulation	Centroiding
Isotope correction at MS1	No	Nomenclature for intact lipid molecule	Yes

Lipid class LPE O-p[M-H]- / For additional separation methods/analytical dimension

Quantitative	Yes	Limit of quantification	S/N ratio
Internal lipid standard(s)	LPE 17:1	Normalization to reference	No
Type of quantification	Internal standard amount	Lipid Quantification Software	LipotypeXplorer
Response correction	No	Batch correction	Normalization by reference material/QC
Type I isotope correction	Yes		

Lipid class LPE[M-H]- / Lipid identification

Lipid class	LPE	MS1 verified by standard	Yes
MS Level	MS1	Background check at MS1	Yes
Identification level	Species level	Check isomer overlap	Yes
Polarity mode	Negative	Lipid Identification Software	LipotypeXplorer
Type of negative (precursor)ion	[M-H]-	Data manipulation	Centroiding
Isotope correction at MS1	Type 2	Nomenclature for intact lipid molecule	Yes

Lipid class LPE[M-H]- / For additional separation methods/analytical dimension

Quantitative	Yes	Limit of quantification	S/N ratio
Internal lipid standard(s)	LPE 17:1	Normalization to reference	No
Type of quantification	Internal standard amount	Lipid Quantification Software	LipotypeXplorer
Response correction	No	Batch correction	Normalization by reference material/QC
Type I isotope correction	Yes		

Lipid class PC[M+CH3COO]- / Lipid identification

Lipid class	PC	MS1 verified by standard	Yes
MS Level	MS1, MS2	MS2 verified by standard	Yes
Identification level	Molecular species level	Background check at MS1	Yes
Polarity mode	Negative	Background check at MS2	Yes
Type of negative (precursor)ion	[M+CH3COO]-	Check isomer overlap	Yes
How many fragments used for ID	2 fragments	Lipid Identification Software	LipotypeXplorer
Fragment ion 1	fatty acyl ion	Data manipulation	Centroiding
Fragment ion 2	fatty acyl ion	Nomenclature for intact lipid molecule	Yes
Isotope correction at MS1	Type 2	Nomenclature for fragment ions	Yes
Isotope correction at MS2	Type 2		

Lipid class PC[M+CH3COO]- / For additional separation methods/analytical dimension

Quantitative	Yes	Limit of quantification	S/N ratio
Internal lipid standard(s)	PC 17:0/17:0	Normalization to reference	No
Type of quantification	Internal standard amount	Lipid Quantification Software	LipotypeXplorer
Response correction	No	Batch correction	Normalization by reference material/QC
Type I isotope correction	Yes		

Source Data (uncropped gels)

SFigure 8

a

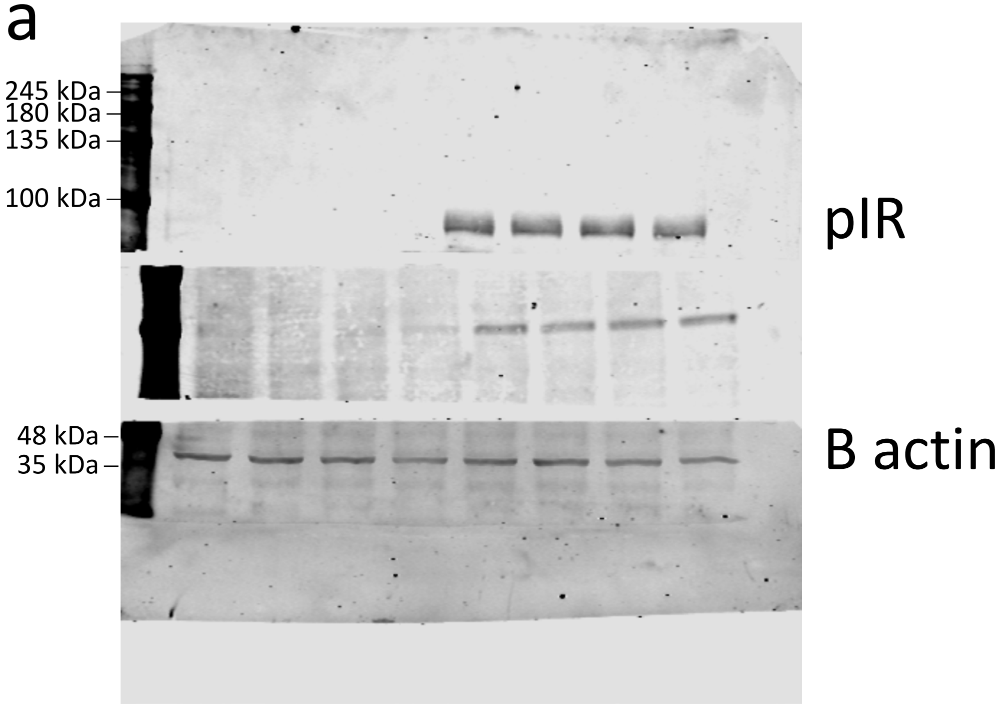

b/c

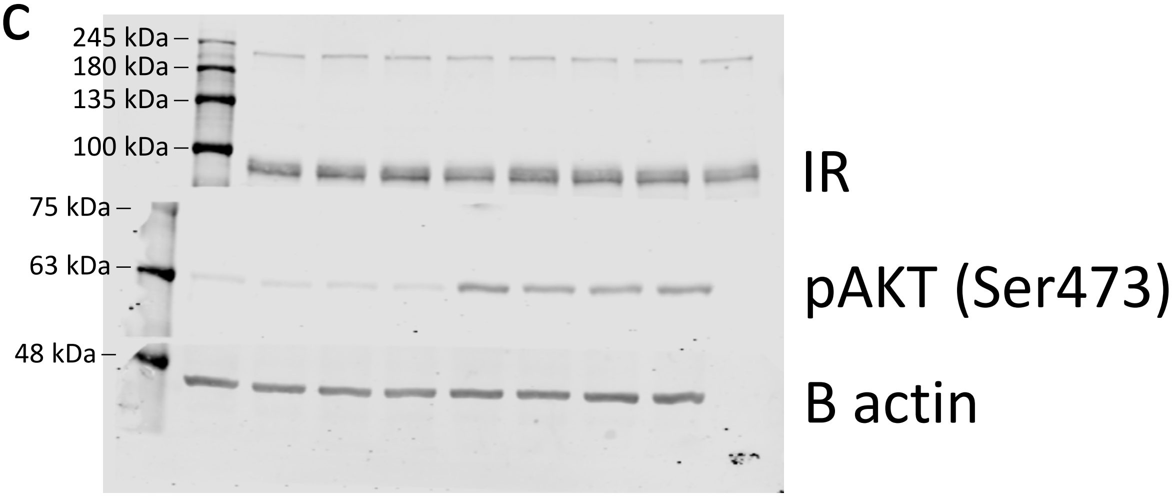

SFigure 8

d

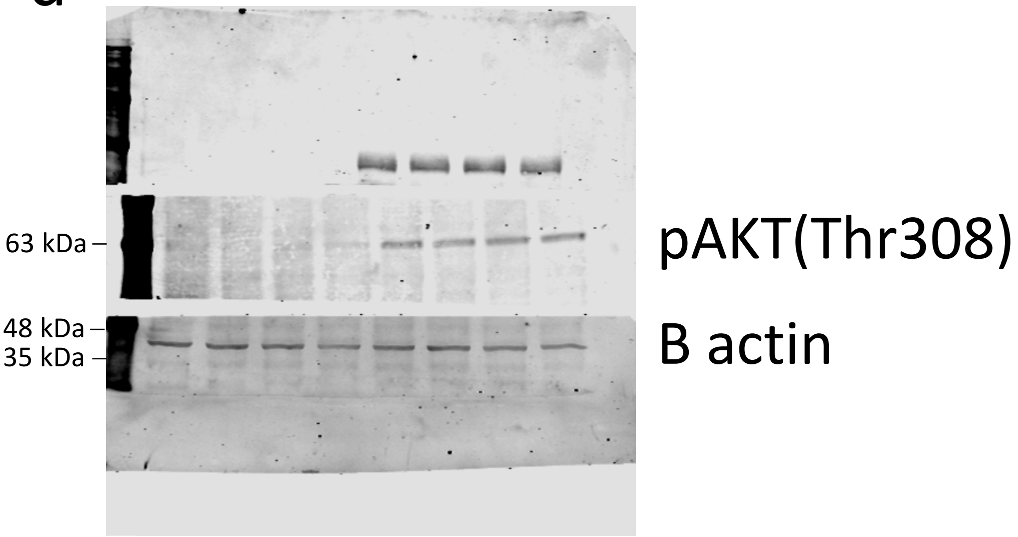

63 kDa
48 kDa
35 kDa
pAKT(Thr308)
B actin

e

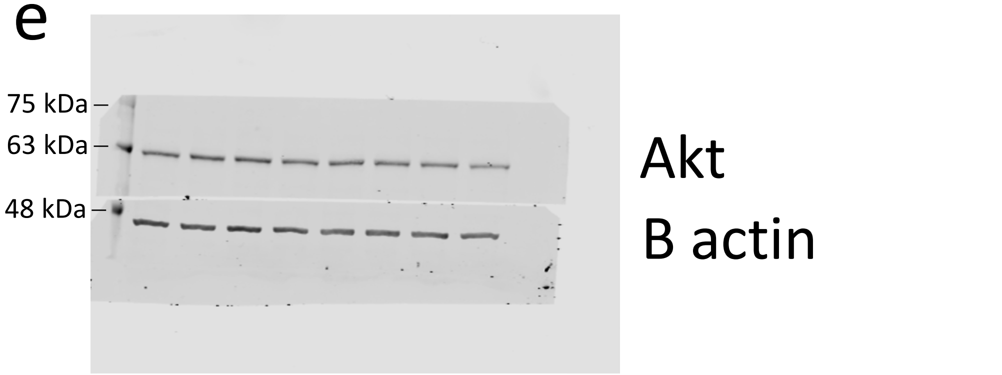

75 kDa
63 kDa
48 kDa
Akt
B actin

SFigure 9

a b c d

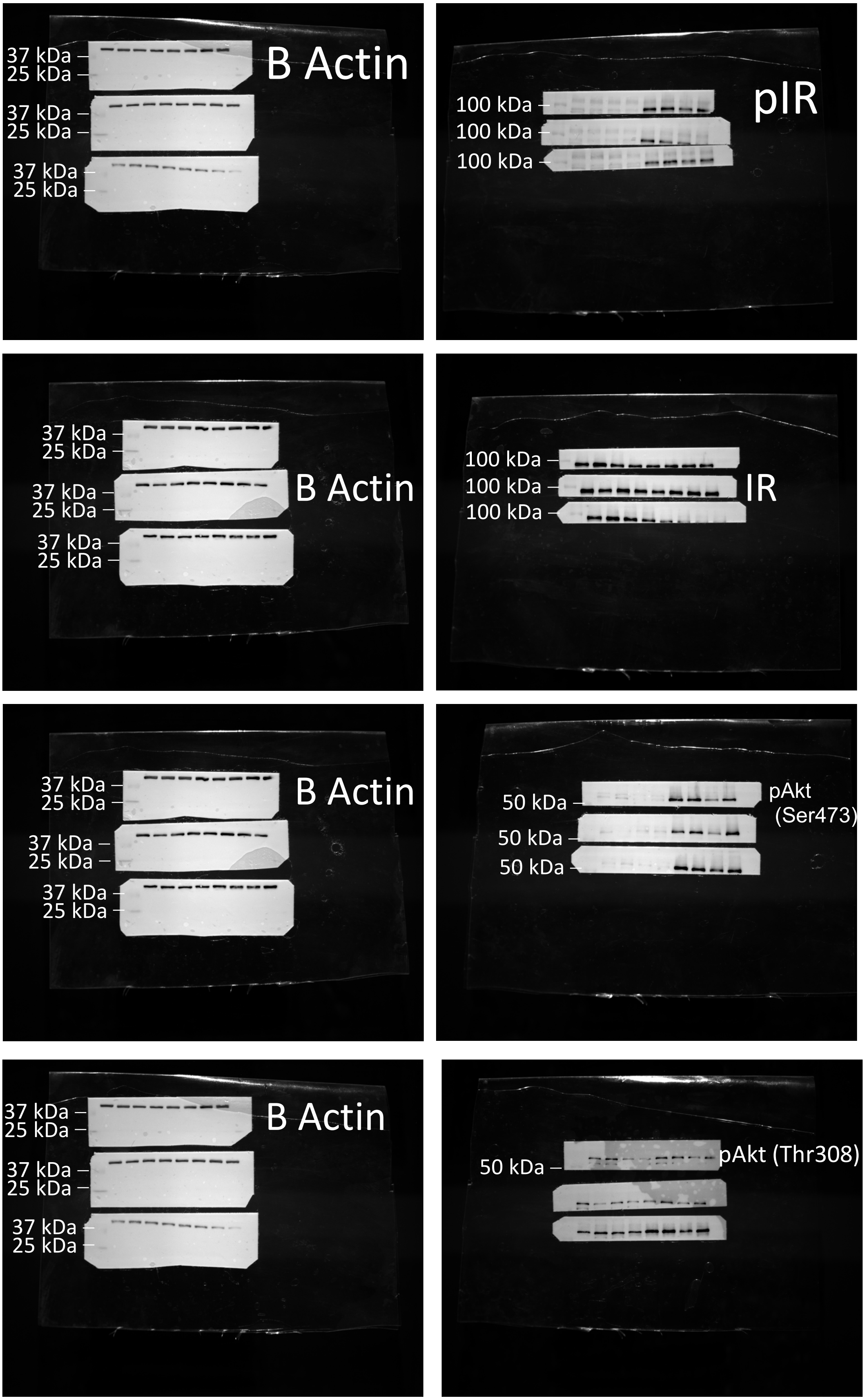

SFigure 9

e

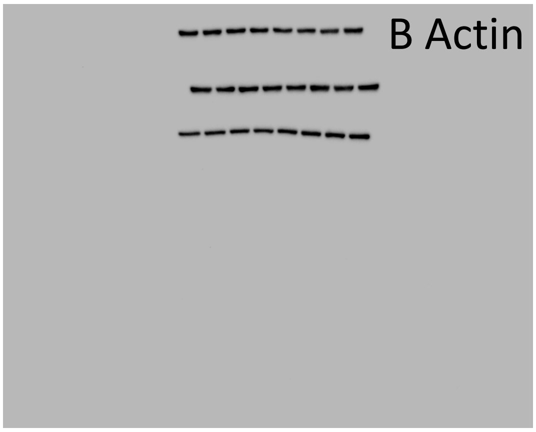

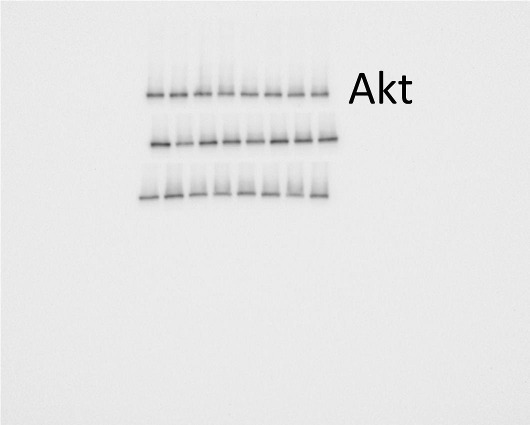

e (with markers)

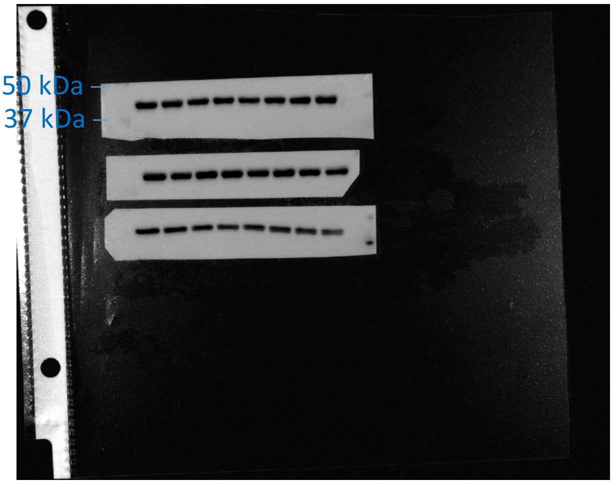

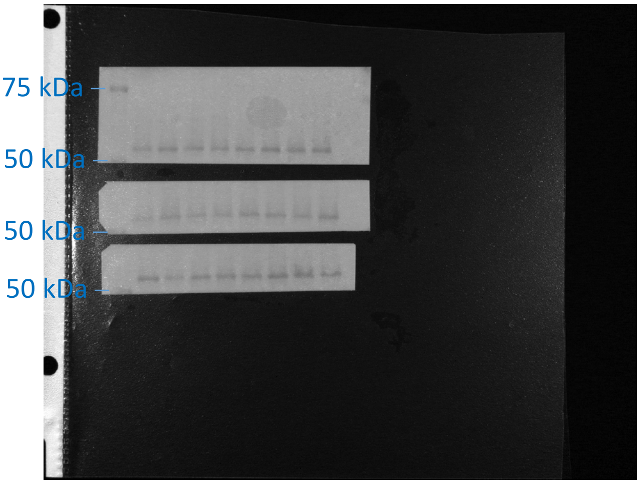

SFigure 10

a

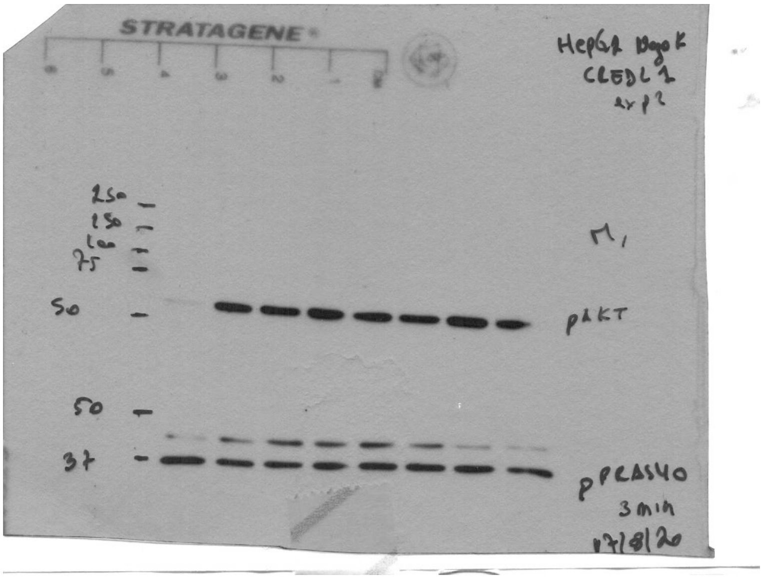

pAKT

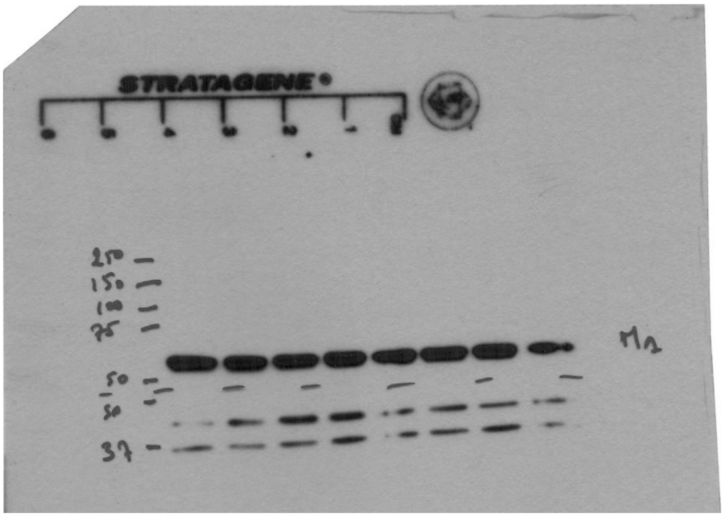

Total AKT

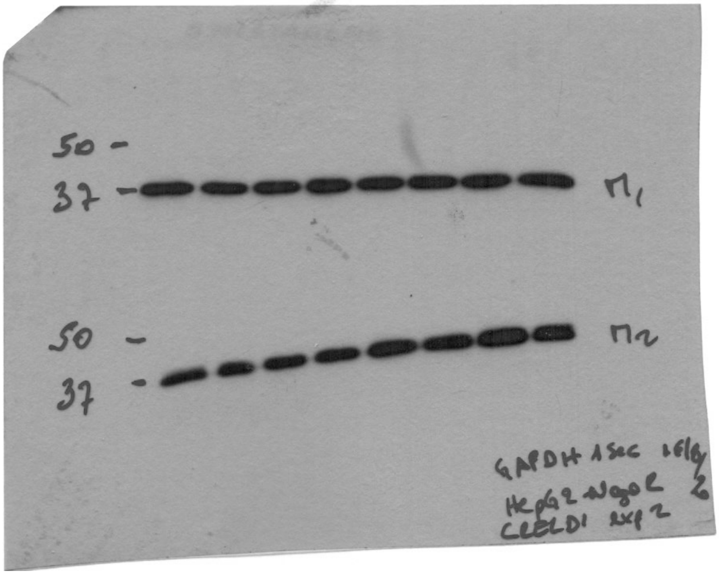

GAPDH

SFigure 10

b

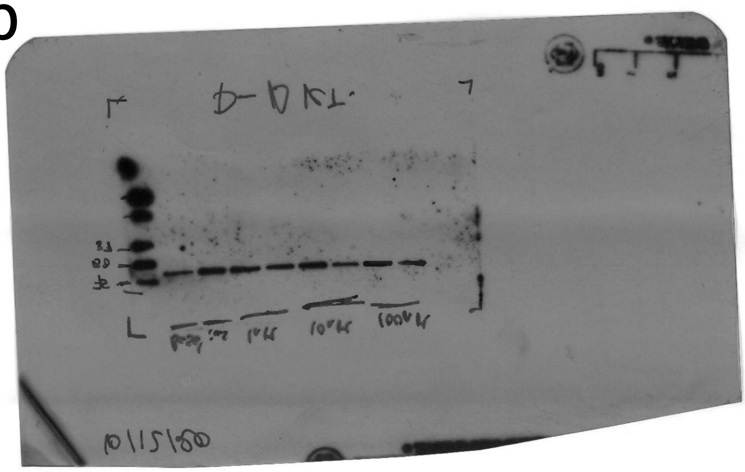

pAkt(Ser473)

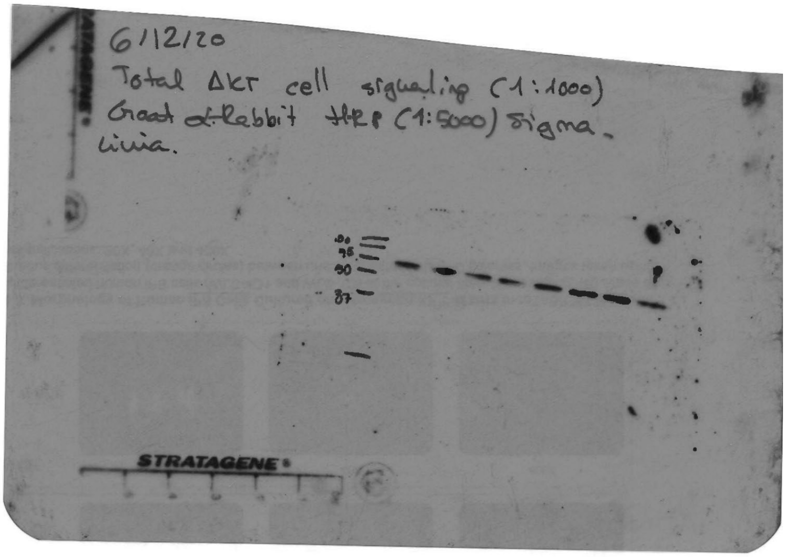

AKT

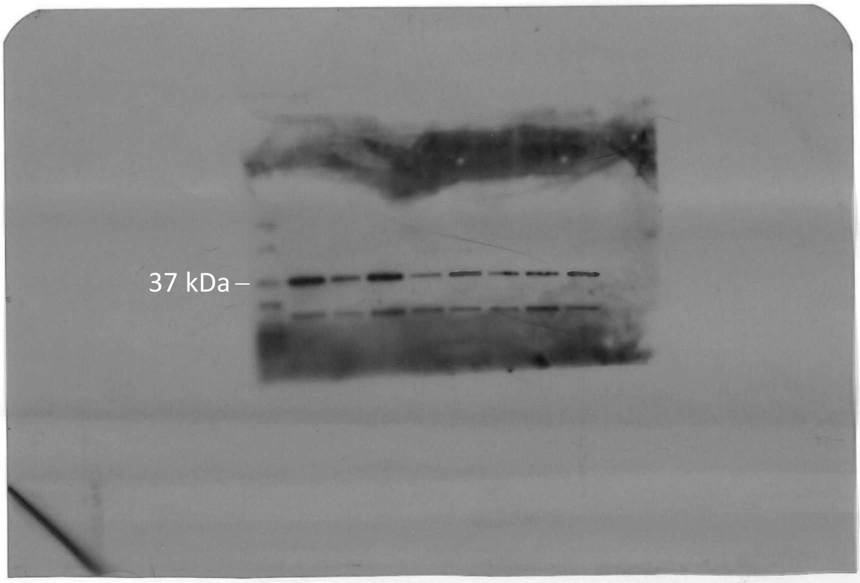

GAPDH
