# Supplementary material for: Identification of biomarkers for glycaemic deterioration in type 2 diabetes
Source: Nat Commun. 2023 May 3;14:2533. doi: 10.1038/s41467-023-38148-7 (PMC10156700; doi:10.1038/s41467-023-38148-7)
Supplement: Supplementary file 3 — Description to additional Supplementary Information [file 41467_2023_38148_MOESM3_ESM.pdf]

Supplementary Data 1. Cox proportional hazard models for the lipids
Supplementary Data 2. Cox proportional hazard models for the proteins
Supplementary Data 3. Included lipid species with SwissLipids names
Supplementary Data 4. Mean and standard deviation for TAGs in DCS, GoDARTS and ANDIS
