# Supplementary material for: Identification of biomarkers for glycaemic deterioration in type 2 diabetes
Source: Nat Commun. 2023 May 3;14:2533. doi: 10.1038/s41467-023-38148-7 (PMC10156700; doi:10.1038/s41467-023-38148-7)
Supplement: Supplementary file 8 — Reporting Summary [file 41467_2023_38148_MOESM8_ESM.pdf]

nature portfolio

Corresponding author(s): Guy A. Rutter, Ewan Pearson, Leen 't Hart

Last updated by author(s): 12-04-2022



# Reporting Summary

Nature Portfolio wishes to improve the reproducibility of the work that we publish. This form provides structure for consistency and transparency in reporting. For further information on Nature Portfolio policies, see our Editorial Policies and the Editorial Policy Checklist.

## Statistics

For all statistical analyses, confirm that the following items are present in the figure legend, table legend, main text, or Methods section.

| n/a | Confirmed | |
|---|---|---|
| ☐ | ☒ | The exact sample size (*n*) for each experimental group/condition, given as a discrete number and unit of measurement |
| ☐ | ☒ | A statement on whether measurements were taken from distinct samples or whether the same sample was measured repeatedly |
| ☐ | ☒ | The statistical test(s) used AND whether they are one- or two-sided<br>*Only common tests should be described solely by name; describe more complex techniques in the Methods section.* |
| ☐ | ☒ | A description of all covariates tested |
| ☐ | ☒ | A description of any assumptions or corrections, such as tests of normality and adjustment for multiple comparisons |
| ☐ | ☒ | A full description of the statistical parameters including central tendency (e.g. means) or other basic estimates (e.g. regression coefficient) AND variation (e.g. standard deviation) or associated estimates of uncertainty (e.g. confidence intervals) |
| ☒ | ☐ | For null hypothesis testing, the test statistic (e.g. *F*, *t*, *r*) with confidence intervals, effect sizes, degrees of freedom and *P* value noted<br>*Give P values as exact values whenever suitable.* |
| ☒ | ☐ | For Bayesian analysis, information on the choice of priors and Markov chain Monte Carlo settings |
| ☒ | ☐ | For hierarchical and complex designs, identification of the appropriate level for tests and full reporting of outcomes |
| ☒ | ☐ | Estimates of effect sizes (e.g. Cohen's *d*, Pearson's *r*), indicating how they were calculated |

*Our web collection on statistics for biologists contains articles on many of the points above.*

## Software and code

Policy information about availability of computer code

| | |
|---|---|
| Data collection | Summary statistics of lipidomic, proteomic and metabolomic data will be available from an interactive Shiny dashboard available upon publication. |
| Data analysis | R code used is available via GitHub: https://github.com/roderickslieker/RHAPSODY (DOI: 10.5281/zenodo.7529655). |

For manuscripts utilizing custom algorithms or software that are central to the research but not yet described in published literature, software must be made available to editors and reviewers. We strongly encourage code deposition in a community repository (e.g. GitHub). See the Nature Portfolio guidelines for submitting code & software for further information.

## Data

Policy information about availability of data

All manuscripts must include a data availability statement. This statement should provide the following information, where applicable:

- Accession codes, unique identifiers, or web links for publicly available datasets
- A description of any restrictions on data availability
- For clinical datasets or third party data, please ensure that the statement adheres to our policy

Discovery cohorts Summary statistics of lipidomic, proteomic and metabolomic data is available from a Shiny dashboard available from: https://rhapdata-app.vital-it.ch. The generated metabolomic, lipidomic and proteomic data in DCS, GoDARTS and ANDIS are considered sensitive patient data and can therefore not be publicly available in compliance with the European privacy regulations governed by GDPR and according to limitations included in the informed consents signed by

the study participants. Please see below information on how to request the data.
Metabolomics and lipidomics (DCS, GoDARTS, ANDIS) data are available upon request by contacting the senior authors (dr. LM 't Hart (lmthart@lumc.nl), prof. dr. E.R. Pearson (E.Z.Pearson@dundee.ac.uk), prof. dr. ir. JWJB Beulens (j.beulens@amsterdamumc.nl) and. dr. G. Rutter (g.rutter@imperial.ac.uk). Requests should include name and contact details of the person requesting the data, which molecular data and clinical variables are requested and the purpose of requesting the data. Requests will be subject to consideration by the steering committees of the three cohorts (DCS, ANDIS, GoDARTS) and the management board of RHAPSODY. Time frame for a response will be within four months . Data requests under agreement will be considered for purposes of reproducing the data and subject to appropriate confidentiality obligations and restrictions.
DCS and GoDARTS proteomics data: restricted access for the proteomics data can be obtained via the European Genome/Phenome archive under accession number EGAD00010002447 (https://ega-archive.org/datasets/EGAD00010002447). Requests via EGA will be forwarded to the corresponding authors and subjected to the same procedure and time frame as the metabolomic and lipidomic data as outlined above.
Replication data: Proteomics data of individuals with incident diabetes described in Gudmundsdottir et al.32 were used for lookups of our protein top hits. Lipid top hits were compared to the lipid data of people with and without diabetes from Fernandez et al.. The GWAS on lipids described by Tabassum et al.80 was used to identify lipid QTLs (https://mqtl.fimm.fi). The GWAS data of Lotta et al.81 was used to identify metabolite QTLs. The GWAS on type 2 diabetes from Mahajan et al.82 was used to find diabetes risk variants.
Functional studies: Source data for functional studies are provided with this paper.

## Human research participants

Policy information about studies involving human research participants and Sex and Gender in Research.

Reporting on sex and gender

No sex specific-analyses were perfomed. Both sexes were included in the analyses.

Population characteristics

Discovery cohorts

Specific details on DCS7, GoDARTS8 and ANDIS9 have been described elsewhere. These cohorts were selected based in part on satisfactory quality control for biomarkers stability in stored samples.

Briefly, the Hoorn Diabetes Care System (DCS) cohort is a prospective cohort with currently over 14,000 individuals with routine care data. The Ethical Review Committee of the VU University Medical Center, Amsterdam approved the study. In 2008-2014, additional blood sampling was done in 5,500 participants, who provided written informed consent. These samples were used for this study. The turbidimetric inhibition immunoassay for haemolyzed whole EDTA blood (Cobas c501, Roche Diagnostics, Mannheim, Germany) was used to measure HbA1c. HDL (mmol/L) was measured enzymatically (Cobas c501, Roche Diagnostics). C-peptide was measured on a DiaSorin Liaison (DiaSorin, Saluggia, Italy).

The Genetics of Diabetes Audit and Research Tayside Study (GoDARTS) is a cohort of ?8,000 patients with T2D. The study was approved by the Tayside Medical Ethics Committee and all individuals provided informed consent. Laboratory measurements were measured in a non-fasted state. C-peptide was measured on a DiaSorin Liaison (DiaSorin, Saluggia, Italy).

In the All New Diabetics in Scania (ANDIS) cohort, people with incident diabetes within Scania County, Sweden were recruited from January 2008 until November 2016 and all participants gave written informed consent. Regional ethics review committee in Lund approved the study. An electro-chemiluminescence immunoassay was used to measure C-peptide on a Cobas e411 (Roche Diagnostics, Mannheim, Germany) or a radioimmunoassay (Human C-peptide RIA; Linco, St Charles, MO, USA; or Peninsula Laboratories, Belmont, CA, USA). The Clinical Chemistry database was used to obtain HbA1c levels.
Validation cohorts

External Replication cohorts
External validation was performed in four external cohorts, ACCELERATE, AGES-Reykjavik, MDC-CC and DESIR. ACCELERATE is a clinical trial aimed at investigating the effect of evacetrapib on major adverse cardiovascular outcomes and has been described elsewhere.11 For the current study we only included the 6,054 individuals in the untreated arm. From this group, we selected 2,978 individuals with type 2 diabetes. In this group, 1003 individuals were excluded that did not have C-peptide levels or HbA1c levels, 72 were excluded because the age at diagnosis was < 35 years, 31 were excluded because they were on insulin at baseline and 22 were excluded because they had > 2 non-insulin glucose-lowering drugs and HbA1c levels > 8.5%. The final set consisted of 1,850 individuals of which 162 reached the primary endpoint.

AGES-Reykjavik is a prospective population-based study from Iceland.12, 13 In fasted blood samples protein levels were measured with the Somalogic platform. At baseline there were 4784 individuals free of diabetes and 654 with type 2 diabetes. Of 2,940 individuals free of diabetes at baseline and with 5-year follow-up information, 112 developed type 2 diabetes. 13 Identified proteins were tested against incident and prevalent type 2 diabetes using logistic regression, adjusted for age and sex.

Malmö Diet and Cancer Cardiovascular Cohort (MDC-CC) is a population-based cohort comprised of people living Malmö.14 Lipids were measured using the Lipotype platform in 3,667 individuals of which 555 developed type 2 diabetes.15 Proteins were measured using the Olink Proseek Multiplex proximity extension assay in 4915 individuals of which 700 developed type 2 diabetes. Identified lipids and proteins were tested against incident diabetes using Cox proportional hazard model adjusted for age, sex and BMI.

DESIR is a prospective population-based cohort comprised of middle-aged European individuals. Metabolomics was measured by Metabolon (Durham, NC).16 Logistic regression adjusted for age, sex and BMI was used to tested for an association between metabolites and prevalent (n = 43) and incident (n = 231) type 2 diabetes versus controls (n = 813).

Functional studies
Human pancreatic islets were purchased from Prodo Labs through the Integrated Islet Distribution Program and with


March 2021

| | |
|---|---|
| | appropriate research consent from organ procurement organizations. Studies in human islets were conducted in compliance with principles of Eli Lilly Bioethics Framework for Human Biomedical Research and approved by the local Bioethics Committee in accordance with internal review board ethical guidelines for use of human tissue. |
| Recruitment | Please see details under "Ethics oversight" |
| Ethics oversight | Discovery cohorts<br>This study the study was conducted in line with the Declaration of Helsinki.61 For DCS, the Ethical Review Committee of the VU University Medical Center, Amsterdam approved the study and written informed consent was obtained. The Tayside Medical Ethics Committee approved the GoDARTS study and participants provided written informed consent. The ANDIS protocol was approved by the Regional Ethical Review Board in Lund, Sweden (584/2006, 2011/354, 2014/198). All participants provided written informed consent.<br><br>Replication cohorts<br>The MDC study was approved by the Regional Ethical Review Board in Lund, Sweden (LU 51/90). All participants provided written informed consent. The DESIR study was approved by the Ethics Committee (CCPPRB) of the Bicêtre Hospital and all participants provided written informed consent. Plasma sample and data used in this study deriving from the ACCELERATE trial (as detailed in Lincoff, AM et al NEJM, 2018) were provided by Eli Lilly pharmaceutical company (data owners of the placebo arm of the trial; co-authors IP, KD, AE).62 The multicentre trial involved 543 centres in 36 countries and for each of them the appropriate national and/or institutional regulatory and ethics boards approved the protocol independently (protocol number of internal approval at Lily, I1V-MC-EIAN-9/19/19). 62 All patients provided written informed consent. Use of existing de-identified samples from the ACCELERATE cohort for the present research is classified as non-human research, and thus IRB approval was not required. The AGES-Reykjavik study was approved by the NBC in Iceland (approval number VSN-00-063), and the National Institute on Aging Intramural Institutional Review Board, and the Data Protection Authority in Iceland. All participants provided written informed consent. Study participants did not receive compensation for any of the studies included.<br><br>Functional studies<br>Human pancreatic islets were purchased from Prodo Labs through the Integrated Islet Distribution Program, and used in Lilly Laboratories Indianapolis, IN, in compliance with the Eli Lilly Bioethics Framework for Human Biomedical Research, with appropriate research consent from the organ procurement organizations. According to regulation 45 CFR 46.of the U.S. Department of Health and Human Services (https://www.hhs.gov) this research is classified as not-human subject research and as such does not require IRB approval. |

Note that full information on the approval of the study protocol must also be provided in the manuscript.

# Field-specific reporting

Please select the one below that is the best fit for your research. If you are not sure, read the appropriate sections before making your selection.

☒ Life sciences ☐ Behavioural & social sciences ☐ Ecological, evolutionary & environmental sciences

For a reference copy of the document with all sections, see nature.com/documents/nr-reporting-summary-flat.pdf

# Life sciences study design

All studies must disclose on these points even when the disclosure is negative.

| | |
|---|---|
| Sample size | Sample size was based on the largest available set of samples for each omics type within the allocated budget. Given the large sample sizes in the three datatypes our study has sufficient power to detect differences between groups. |
| Data exclusions | Samples were only excluded in when they did not failed in the quality control. For lipidomics 2 samples failed QC, for proteomics 12 samples. |
| Replication | Replication was performed in 1) new samples from the same cohort (metabolomics) 2) samples from new cohorts with the same endpoint (proteomics) 3) external cohorts with the incident diabetes endpoint (metabolomics, lipidomics, proteomics). Results of these replications confirmed the findings in the discovery cohorts. |
| Randomization | N/A |
| Blinding | N/A |

# Reporting for specific materials, systems and methods

We require information from authors about some types of materials, experimental systems and methods used in many studies. Here, indicate whether each material, system or method listed is relevant to your study. If you are not sure if a list item applies to your research, read the appropriate section before selecting a response.

## Materials & experimental systems

| n/a | Involved in the study |
|---|---|
| ☐ | ☒ Antibodies |
| ☐ | ☒ Eukaryotic cell lines |
| ☒ | ☐ Palaeontology and archaeology |
| ☐ | ☒ Animals and other organisms |
| ☐ | ☒ Clinical data |
| ☒ | ☐ Dual use research of concern |

## Methods

| n/a | Involved in the study |
|---|---|
| ☒ | ☐ ChIP-seq |
| ☒ | ☐ Flow cytometry |
| ☒ | ☐ MRI-based neuroimaging |

# Antibodies

| | |
|---|---|
| Antibodies used | PDX1 (ab47308, Abcam); AKT: Cell Signalling #9272; phosho(Ser472) AKT #9271; pAkt (Thr308) from cell signaling (9275S); Insulin receptor β from cell signaling (3025S), Phospho-IGF-I Receptor β (Tyr1131)/Insulin Receptor β (Tyr1146) from cell signaling (3021S), and Beta actin from Thermofisher A5441).; anti-insulin, Agilent DAKO, A0564; anti-glucagon (PU-039-UP, Biogenex, Fremont, CA) |
| Validation | As per manucfacturers' websites. |

# Eukaryotic cell lines

Policy information about cell lines and Sex and Gender in Research

| | |
|---|---|
| Cell line source(s) | HEK293-IL18R (Invivogen.com), HepG2 (Professor Axel Kahn, Insitut Cochin, Paris); C3H10T1/2 adipocytes from ATCC, courtesy of Pierre Moffat, McGill University, Montreal |
| Authentication | Lines not routinely authenticated |
| Mycoplasma contamination | Checked routinely using Lonza MycoAlertTM Mycoplasma Detection Kit (10 Tests) (Catalog #: LT07-118). This is a luminescent assay. |
| Commonly misidentified lines (See ICLAC register) | None of the cell lines used feature in the list of known misidentified cell lines (https://iclac.org/databases/cross-contaminations). |

# Animals and other research organisms

Policy information about studies involving animals; ARRIVE guidelines recommended for reporting animal research, and Sex and Gender in Research

| | |
|---|---|
| Laboratory animals | Mouse, C57/BL6, 12-15 weeks; db/db mice 8-12 weeks |
| Wild animals | None |
| Reporting on sex | Only male mice were included and as such no sex-specific analyses were performed. |
| Field-collected samples | None |
| Ethics oversight | Ethics approval for rodent studies was obtained from the UK Home Office, according to the Animals (Scientific Procedures) Act 1986, with local ethical committee (Imperial College AWERB) and under a personal project license (PPL) number PA03F7F07 to I.L., or the Animal Care Committee at the Institut de recherches cliniques de Montréal (J.E.). Animals were maintained in approved institutional animal facilities overseen by qualified veterinary teams, under specific pathogen free conditions. Day to day monitoring of animal wellbeing was performed by facility technicians and by the researchers directly involved in the studies. |

Note that full information on the approval of the study protocol must also be provided in the manuscript.

# Clinical data

Policy information about clinical studies

All manuscripts should comply with the ICMJE guidelines for publication of clinical research and a completed CONSORT checklist must be included with all submissions.

| | |
|---|---|
| Clinical trial registration | *Provide the trial registration number from ClinicalTrials.gov or an equivalent agency.* |
| Study protocol | *Note where the full trial protocol can be accessed OR if not available, explain why.* |
| Data collection | *Describe the settings and locales of data collection, noting the time periods of recruitment and data collection.* |
| Outcomes | *Describe how you pre-defined primary and secondary outcome measures and how you assessed these measures.* |
